# Mapping Dental Care for Children and Adolescents With Rare Diseases: A Brazilian Multicentre Study

**DOI:** 10.1111/cdoe.70029

**Published:** 2025-10-03

**Authors:** Heloisa Vieira Prado, Rayssa Maria Soalheiro de Souza, Gabriella Guerra Freire Gabrich Fonseca, Kamila Rodrigues Junqueira Carvalho, Anna Vitória Mendes Viana Silva, Iasmin Fonseca Tolentino Mascarenhas, Beatriz Rezende Bergo, Hanna Larissa Barbosa Soares, Bárbara Mendes de Jesus, Layanne Ribeiro Ferreira e Sobral, Kélisson Duarte Reis, Késia Lara dos Santos Marques, Fabiana Sodré de Oliveira, Daniella Reis Barbosa Martelli, José Alcides Almeida de Arruda, Benjamin P. J. Fournier, Denise Vieira Travassos, Soraia Macari, Célia Regina Moreira Lanza, Ana Cristina Borges‐Oliveira, Hercílio Martelli‐Júnior, Tarcília Aparecida Silva

**Affiliations:** ^1^ Department of Oral Surgery, Pathology and Clinical Dentistry, School of Dentistry Universidade Federal de Minas Gerais Belo Horizonte Brazil; ^2^ Multiprofessional Integrated Residency in Health Universidade Federal de Minas Gerais Belo Horizonte Brazil; ^3^ Department of Child and Adolescent Oral Health, School of Dentistry Universidade Federal de Minas Gerais Belo Horizonte Brazil; ^4^ Department of Restorative Dentistry, School of Dentistry Universidade Federal de Minas Gerais Belo Horizonte Brazil; ^5^ Oral Medicine and Oral Pathology, School of Dentistry Universidade Estadual de Montes Claros Montes Claros Brazil; ^6^ Postgraduate Program in Dental Sciences, School of Dentistry Universidade Federal de Alfenas Alfenas Brazil; ^7^ Center for Rehabilitation of Craniofacial Anomalies, School of Dentistry Universidade Professor Edson Antônio Velano Alfenas Brazil; ^8^ Department of Pediatric Dentistry, School of Dentistry Universidade Federal de Uberlândia Uberlândia Brazil; ^9^ Postgraduate Program in Primary Care/Health Sciences Universidade Estadual de Montes Claros Montes Claros Brazil; ^10^ Department of Oral Diagnosis and Pathology, School of Dentistry Universidade Federal do Rio de Janeiro Rio de Janeiro Brazil; ^11^ Reference Center for Dental Rare Diseases (O‐Rares), Rothschild Hospital Paris France; ^12^ Department of Oral Biology, School of Dentistry Université de Paris Paris France; ^13^ Centre de Recherche Des Cordeliers, Laboratory of Molecular Oral Pathophysiology, Université de Paris, Inserm, Sorbonne Université Paris France; ^14^ Department of Social and Preventive Dentistry, School of Dentistry Universidade Federal de Minas Gerais Belo Horizonte Brazil

**Keywords:** dental assistance, dental care for children, oral health, orphan diseases, paediatrics, rare diseases

## Abstract

**Objectives:**

To describe the landscape of dental care provided by specialised centres for children and adolescents with rare diseases (RDs) in the state of Minas Gerais, southeastern Brazil.

**Methods:**

A retrospective cross‐sectional study was conducted involving individuals aged 0–18 years with a confirmed diagnosis of a RD who received care at five specialised dental centres. Data on the diagnosis, age at first dental appointment, frequency of annual visits and travel distance from home to treatment centre were analysed using descriptive and inferential statistics.

**Results:**

A total of 1057 individuals with 244 different RDs were identified. Most were boys (54.9%). The average age at the first dental appointment was 8.52 years. Haematological diseases were the most prevalent (38.9%). The average travel distance for treatment was 99.1 km, with individuals from the Jequitinhonha region traveling the farthest (526.3 km). The average number of annual dental visits was 2.4. Patients with craniofacial syndromes accessed care earlier (average: 3.6 years) and had more frequent follow‐up appointments (average: 4.8 visits/year). Significant regional disparities were found in age at first appointment (*p* < 0.001), travel distance (*p* < 0.001) and frequency of visits (*p* = 0.002).

**Conclusions:**

Children and adolescents with RDs had delayed initiation of dental care, low follow‐up rates and substantial travel burdens. The concentration of specialised centres in the state capital underscores the need for policy reforms to improve equitable access, particularly for patients in remote areas.

## 1 Introduction

Rare diseases (RDs) comprise a heterogeneous group of disorders that may affect any organ system and are primarily characterised by the low prevalence in the general population [1, 2]. A disease is classified as rare when its prevalence does not exceed 65 cases per 100,000 individuals [1, 3, 4]. Approximately 80% of such conditions have a genetic aetiology [1, 3, 4]. Between 6000 and 8000 RDs have been identified thus far, with new entities continuously being described in the literature. Although global prevalence estimates remain imprecise, RDs are collectively estimated to affect between 300 and 400 million individuals throughout the world [2, 4]. In Brazil, approximately 13 million people are estimated to be living with RDs, corresponding to a rate of 6.5 cases per 10 000 individuals [2, 5].

Many RDs require continuous medical care, particularly debilitating or degenerative diseases [2]. Individuals with RDs often have impaired physical, mental, sensory and behavioural functions, which compromise autonomy and overall quality of life [1, 6]. Given these complexities, multidisciplinary care is essential. Orofacial anomalies are common in many RDs, increasing vulnerability to oral health problems [7, 8, 9]. These conditions negatively affect oral health‐related quality of life not only from a psychosocial standpoint but also by influencing treatment outcomes and access to care [10]. Consequently, the inclusion of dentists on multidisciplinary teams is crucial to ensuring comprehensive management [11, 12]. Nonetheless, scientific evidence on dental care for individuals with RDs remains scarce both in Brazil and internationally. Previous studies have identified significant barriers to accessing dental services, further compounding oral health challenges in this population [12, 13, 14, 15].

The 2014 National Policy for Comprehensive Care for People with Rare Diseases marked a milestone in the recognition of RDs within Brazil's universal healthcare system, which is organised as a tiered referral system encompassing primary to specialised care [3, 4]. The public healthcare system is structured to ensure universal access to healthcare, with primary care serving as the gateway and core provider of patient care [16]. Depending on the severity of the condition and individual needs, dental care may be delivered through primary care services for preventive measures and less complex procedures. When such services are unable to provide the necessary interventions, patients are referred to specialised care through a formalised referral and counter‐referral system [17].

Specialised dental centres play a crucial role in reducing barriers to oral healthcare for individuals with RDs, particularly in countries characterised by pronounced social and geographic disparities [15]. In Latin America, the absence of standardised definitions and limited epidemiological data further complicates healthcare planning. Recent recommendations emphasise the need for uniform definitions, regional research collaborations and the establishment of a comprehensive RD registry [2]. In Brazil, individuals with RDs face restricted access to dental centres [15, 17], with data on paediatric populations particularly limited. Addressing these gaps through targeted policies and the expansion of specialised centres is essential to reducing health inequities.

The aim of the present study was to describe the landscape of dental care provided at specialised centres for children and adolescents with RDs in the state of Minas Gerais, which is located in southeastern Brazil.

## 2 Methods

### 2.1 Study Design, Setting and Ethical Approval

A cross‐sectional study was developed at five publicly funded dental centres that provide specialised care for individuals with RDs in the state of Minas Gerais, Brazil. The study was reported following the guidelines of the Strengthening the Reporting of Observational Studies in Epidemiology (STROBE statement) [18]. Ethical approval was obtained from the Human Research Ethics Committees of the Universidade Federal de Minas Gerais (UFMG) (no. 63051222.0.0000.5149), Universidade Professor Edson Antônio Velano (no. 67606822.1.0000.5143) and Universidade Federal de Uberlândia (UFU) (no. 36046920.0.0000.5152). The study was conducted in accordance with the principles of the Declaration of Helsinki, and written informed consent was obtained from all participants.

### 2.2 Study Population and Data Collection

Children and adolescents diagnosed with RDs who received care at one of the five participating dental centres were included. The inclusion criteria were age 0–18 years at the time of the first dental appointment and a diagnosis of an RD confirmed by a specialist and listed in the Orphanet database (https://www.orpha.net). Individuals were excluded if documentation was insufficient to confirm the diagnosis or dental treatment history, if the diagnosis was unclear or not aligned with Orphanet definitions, or if data on the residential address were missing.

A standardised data collection form was collaboratively developed by the research team. Three examiners (H.V.P., H.M.‐J. and T.A.S.) underwent a training and calibration process that included training sessions and consensus meetings to ensure consistency in data entry and interpretation to standardise the understanding of all variables and definitions used. Data collection involved a retrospective review of both electronic and physical medical records from the participating centres. The following variables were collected: (i) demographic data: date of birth and date of first dental appointment (used to calculate age at first dental appointment), sex (male/female) and residential address (city and regional division); (ii) clinical information: diagnosis of RD and age at first dental appointment (years); (iii) healthcare access indicators: average number of annual appointments and travel distance to the nearest specialised centre (km), calculated using the Google Maps API (https://www.google.com.br/maps/preview) based on the shortest driving route between each patient's residence and the corresponding specialised dental centre (File S1).

Residential addresses were classified according to the official regional division of the state of Minas Gerais, as defined by the Brazilian Institute of Geography and Statistics (https://www.mg.gov.br/pagina/geografia). The state was divided into 12 geographic regions based on economic, social and cultural characteristics: North of Minas Gerais, Northwest of Minas Gerais, Central Mineira, Belo Horizonte region, Jequitinhonha, Vale do Mucuri, Triângulo Mineiro/Alto Paranaíba, West of Minas Gerais, Vale do Rio Doce, Zona da Mata, South/Southwest of Minas Gerais and Campo das Vertentes.

RDs were grouped into categories based on shared pathophysiological characteristics, as proposed by Friedlander et al. [19] This classification was selected due to its relevance to the dental context, as it enables the identification of syndromes with oral manifestations and systemic conditions that exert an influence on dental care. Each category reflects a distinct pathophysiological domain, facilitating more systematic comparisons and a more focused discussion of the dental needs associated with different disease profiles. The following were the 23 categories of RDs considered in this study: haematological diseases; genetic diseases; autoimmune and autoinflammatory disorders; bone diseases; non‐odontogenic tumours; syndromes with oral and maxillofacial manifestations; central nervous system disorders with motor/cognitive impairment; odontogenic tumours; liver diseases; metabolic diseases; vascular diseases; dermatological diseases; jaw cysts; amelogenesis imperfecta and odontodysplasia; disorders affecting somatic and cognitive development; diseases of brain development and intellectual disability; renal and urological diseases; neuromuscular diseases; neurodegenerative conditions; heart diseases; endocrine diseases; gastrointestinal diseases; and ophthalmological disorders.

### 2.3 Data Analysis

The data were tabulated using Microsoft Office Excel 2019 (Microsoft, Redmond, WA, USA). The Statistical Package for the Social Sciences (SPSS) (IBM SPSS Statistics for Windows, version 25.0, Armonk, NY: IBM Corp.) was used for the statistical analysis of the data. The Kolmogorov–Smirnov test was used to determine the normality of the data, which revealed a non‐normal distribution. Descriptive statistics were performed. Comparisons between groups were conducted using the Kruskal–Wallis test followed by Dunn's *post hoc* test. The chi‐squared test was used for the analysis of categorical data. The level of significance was set at 5% (*p* < 0.05). Graphs and visualisations were generated using the R software (R Core Team, Vienna, Austria) within the RStudio environment (RStudio, Boston, MA, USA), employing the ggplot2 library [20].

## 3 Results

A total of 1212 individuals were initially identified, 155 of whom were excluded for the following reasons: 95 cases in which information on the RD could not be retrieved from the Orphanet database and 60 due to incomplete medical records. Thus, 1057 individuals were included, the majority of whom (*n* = 718; 67.9%) resided in metropolitan Belo Horizonte. Mean age at the first dental appointment was 8.52 ± 5.13 years. Mean distance travelled to the treatment centre was 99.1 ± 145.8 km, with individuals from the Jequitinhonha region travelling the farthest (526.3 ± 168.1 km). The mean number of dental visits per year was 2.35 ± 2.66. Table 1 depicts the five participating specialised dental treatment centres located in the North, South, Triângulo Mineiro and metropolitan Belo Horizonte regions, along with the number of RD cases treated in each region.

**TABLE 1:** Distribution of children and adolescents with rare diseases (RDs) receiving dental care at five specialised centres in Minas Gerais, Brazil.

Specialised centre (coordinates: latitude and longitude)	Region of Minas Gerais	Total of children and adolescents with RDs (*n*, %)	Total of RDs (*n*, %)
HC‐UFMG^a^ (−19.924506; −43.928151)	Belo Horizonte	730 (69)	175 (57.3)
SD‐UFMG^b^ (−19.872396; −43.972685)	Belo Horizonte	211 (20)	76 (25)
SD‐UFU^c^ (−18.881950; −48.260010)	Triângulo Mineiro/Alto Paranaíba	22 (2)	16 (5.2)
Unimontes^d^ (−16.717573; −43.850965)	North	47 (4.5)	20 (6.5)
Pro‐Sorriso^e^ (−21.447329, −46.003287)	South/Southwest	47 (4.5)	18 (6)
Total	—	1057 (100)	305 (100)

^a^ Special Dental Diagnosis and Treatment Service, Hospital das Clínicas, Universidade Federal de Minas Gerais (a hospital affiliated with a public university).

^b^ School of Dentistry, Universidade Federal de Minas Gerais (includes the clinic of oral medicine and pathology, the clinic for children and adolescents with disabilities, the clinic for individuals with cleft lip and palate, and the clinic for individuals with odontogenesis defects; public university).

^c^ School of Dentistry, Universidade Federal de Uberlândia (Public University).

^d^ Oral Pathology and Oral Medicine Service, School of Dentistry, Universidade Estadual de Montes Claros (public university).

^e^ Craniofacial Anomalies Rehabilitation Center, Pro‐Sorriso Center, Universidade Professor Edson Antônio Velano (a centre accredited by the public health system).

**FIGURE 1:**
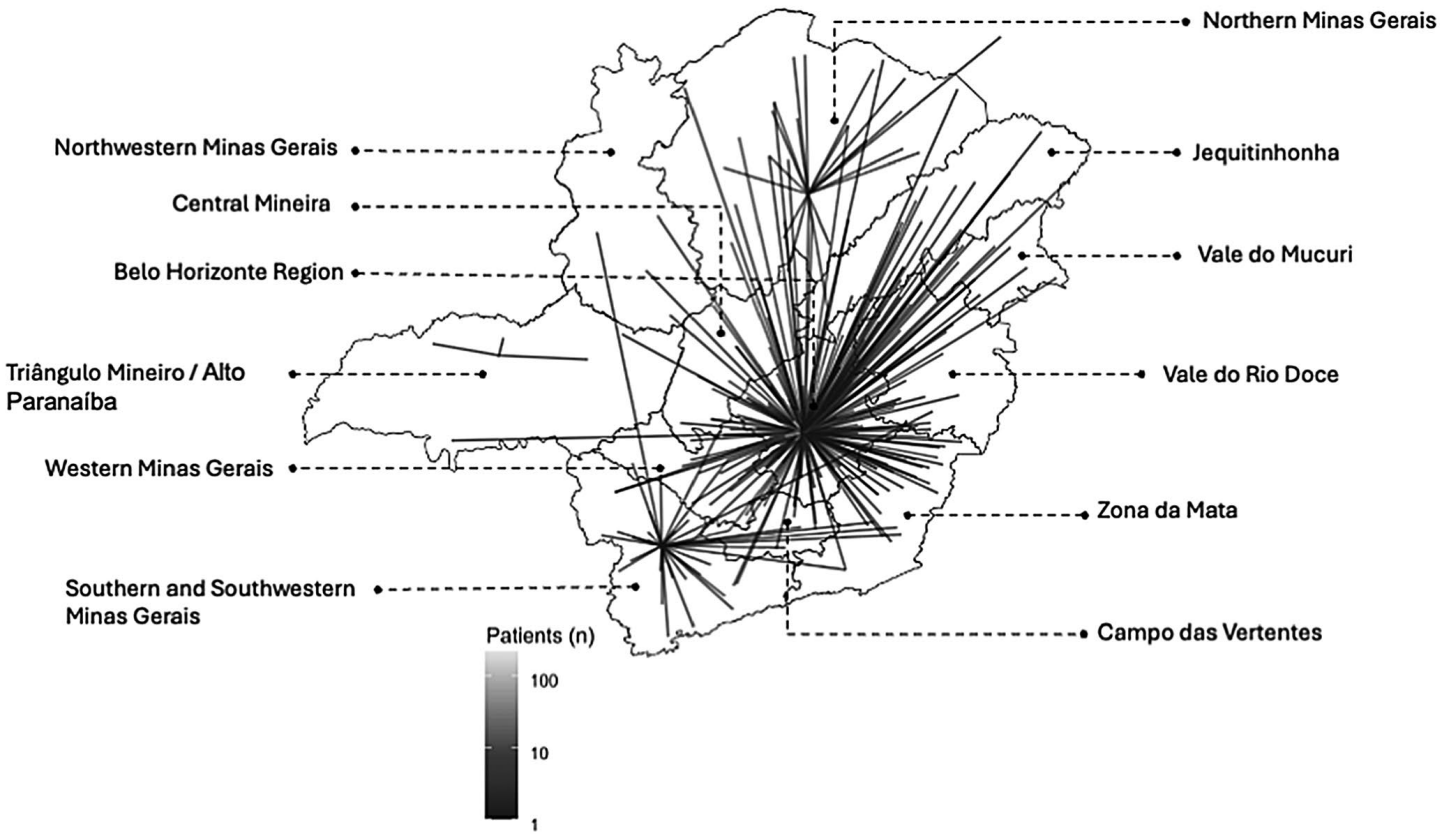
Routes from residences to specialised dental treatment centres for individuals with rare diseases in the state of Minas Gerais, southeastern Brazil.

Table 2 displays the distribution of patients across the specialised centres, along with data on age at first appointment, sex, travel distance and annual dental visits. Among the centres, the hospital affiliated with UFMG had the largest number of patients (*n* = 730; 69%). Statistically significant differences were found among centres with regard to age at first appointment (*p* < 0.001), travel distance (*p* < 0.001) and the number of annual dental visits (*p* < 0.001). Patients treated at the Pro‐Sorriso Center and the School of Dentistry of UFU had the lowest median ages at the first appointment (1 and 2 years, respectively). The Pro‐Sorriso Center also had the highest number of annual visits (mean: 4.53 ± 4.37). The Jequitinhonha region had the greatest geographical barrier to access, with a mean travel distance of 526.3 ± 168.1 km (Figure 1).

**TABLE 2:** Comparison across treatment centres regarding age at first dental consultation, travel distance, and number of annual visits (*n* = 1057).

Specialized centre	*n* (%)	Age (years) at first consultation^‡^	*p* *	Sex, *n* (%)		*p* **	Distance to specialized centre in km^‡^	*p* *	Annual visits^‡^	*p* value*
				Female	Male					
HC‐UFMG^1^	730 (69)	8, 8.55 ± 4.8, 0–18^a^	**< 0.001**	336 (46)	394 (54)	0.332	36.8, 108.8 ± 154.2, 1.7–826^a^	**< 0.001**	1, 2.73 ± 2.7, 1–36^a^	**< 0.001**
SD‐UFMG^2^	211 (20)	10, 9.97 ± 5.2, 0–18^a^		87 (41.2)	124 (58.8)		8.1, 58.8 ± 104.9, 8.1–699^a^		1, 2 ± 1.7,1–12^a^	
Unimontes^3^	22 (2)	10, 9.64 ± 5.26, 0–18^a^		26 (55.3)	21 (44.7)		105, 114.27 ± 137.67, 2.5–756^a^		2, 2.23 ± 1.31, 1–6^a^	
SD‐UFU^4^	47 (4.5)	2, 3.05 ± 3.49, 0–12^b^		11 (50)	11 (50)		5.6, 18.6 ± 38.4, 5.6–149^b^		3, 2.68 ± 1.21,1–6^b^	
Pro‐sorriso^5^	47 (4.5)	1, 3.72 ± 4.85, 0–17^b^		18 (38.3)	29 (61.7)		107, 168.37 ± 159.24, 2.3–654^ab^		2, 4.53 ± 4.37, 1–20^a^	

*Note:* Superscript letters indicate groupings based on Dunn's *post hoc* analysis. Specialised centres sharing the same letter do not differ significantly (*p* ≥ 0.05), whereas those with different letters show a statistically significant difference (*p* < 0.05).

^‡^ Median, mean ± SD, and range.

* Kruskal–Wallis test.

** Chi‐squared test.

^1^ Special Dental Diagnosis and Treatment Service, Hospital das Clínicas, Universidade Federal de Minas Gerais (a hospital affiliated with a public university).

^2^ School of Dentistry, Universidade Federal de Minas Gerais (includes the clinic of oral medicine and pathology, the clinic for children and adolescents with disabilities, the clinic for individuals with cleft lip and palate, and the clinic for individuals with odontogenesis defects; public university).

^3^ School of Dentistry, Universidade Federal de Uberlândia (public university).

^4^ Oral Pathology and Oral Medicine Service, School of Dentistry, Universidade Estadual de Montes Claros (public university).

^5^ Craniofacial Anomalies Rehabilitation Center, Pro‐Sorriso Center, Universidade Professor Edson Antônio Velano (a centre accredited by the public health system).

Haematological diseases were the most prevalent, accounting for 38.9% (*n* = 410) of all cases, with acute lymphoblastic leukaemia as the most frequent diagnosis (*n* = 161; 15.3%) (File S2). Disease distribution varied across the specialised centres: the UFMG hospital predominantly managed haematological conditions (*n* = 409; 99.8%), the Pro‐Sorriso Center concentrated the majority of cases with syndromes involving oral and maxillofacial manifestations (*n* = 27; 57.4%) and Universidade Estadual de Montes Claros (Unimontes) primarily treated vascular diseases (*n* = 13; 54.2%). Genetic diseases were the leading category at the UFMG School of Dentistry (*n* = 30; 14.2%) and UFU School of Dentistry (*n* = 8; 36.4%) (File S3).

Patients diagnosed with syndromes involving the maxillofacial complex accessed care at the youngest age (mean: 3.56 years) and required the most follow‐up visits (mean: 4.8 visits/year). In contrast, individuals with odontogenic tumours and neurodegenerative diseases had the lowest visit frequencies (mean: 1.5 and 1.5, respectively). Statistically significant differences were found among RD groups with regard to age at first dental appointment (*p* < 0.001) and annual number of visits (*p* = 0.002). Although no significant difference in travel distance was found among disease groups (*p* = 0.077), patients with dermatological diseases travelled the longest distances (mean: 152.5 ± 177.8 km) to access dental care (Table 3).

**TABLE 3:** Comparison among rare disease groups regarding age at first consultation, travel distance, and annual number of consultations (*n* = 1057).

Rare diseases	*n* (%)	Age (years) at first consultation^‡^	*p* *	Sex, *n* (%)		*p* **	Distance to specialised centre (km)^‡^	*p* *	Annual visits^‡^	*p* *
				Female	Male					
Haematological diseases	410 (38.9)	9, 8.7 ± 4.6, 0–18^a^	**< 0.001**	169 (41.2)	241 (58.8)	0.220	40.9, 111.6 ± 149.4, 1.7–826	0.077	1, 2.4 ± 3.3, 1–36^a^	**0.002**
Genetic diseases	94 (9)	6, 6.78 ± 5.1, 0–17^a^		50 (53.2)	44 (46.8)		24.4, 104.2 ± 152.9, 1.7–607		2, 2 ± 1.6, 1–9^a^	
Autoimmune and autoinflammatory diseases	64 (6.1)	9, 8.8 ± 5.8, 0–17^a^		31 (48.4)	33 (51.6)		36.8, 99.1 ± 149.4, 1.7–670		1, 2.3 ± 4.2, 1–33^a^	
Bone diseases	60 (5.7)	9, 9.3 ± 5.3, 0–18^b^		26 (43.3)	34 (56.7)		27.3, 78.9 ± 119.9, 1.7–496		2, 3.3 ± 4.4, 1–25^a^	
Non‐odontogenic tumours (benign and malignant)	58 (5.5)	7, 8.1 ± 4.6, 0–18^b^		32 (55.2)	26 (44.8)		24.1, 91.6 ± 158.6, 1.7–826		1, 1.9 ± 2, 1–14^a^	
Syndromes with oral and maxillofacial manifestations	51 (4.7)	1, 3.56 ± 4.8, 0–16^c^		21 (44)	29 (56)		58.7, 117.7 ± 144.2, 1.7–654		3, 4.8 ± 5.2, 1–26^a^	
Disease with motor/cognitive expression of the central nervous system	30 (2.7)	6, 6.75 ± 3.8, 1–13^a^		14 (46.7)	16 (53.3)		22, 103.1 ± 193, 1.7–645		1, 1.8 ± 1.49, 1–7^a^	
Odontogenic tumours (benign and malignant)	26 (2.5)	14.5, 14.4 ± 3.4, 2–18^b^		10 (38.5)	16 (61.5)		20.3, 61.8 ± 69.7, 2.5–218		1, 1.5 ± 1.4, 1–6^b^	
Liver diseases	24 (2.3)	10.5, 9.7 ± 5.2, 0–18^a^		13 (54.2)	11 (45.8)		36.8, 134.5 ± 190.9, 1.7–645		2, 2.5 ± 2.1, 1–8^a^	
Metabolic diseases	24 (2.3)	8.5, 9 ± 5.13, 0–18^a^		8 (33.3)	16 (66.7)		20.9, 48.9 ± 62.5, 1.7–248		2, 2 ± 1.6, 1–8^a^	
Vascular diseases	24 (2.3)	8.5, 8.4 ± 4.3, 1–16^a^		14 (58.3)	10 (41.7)		5.3, 87.6 ± 162.7, 1.7–612		2, 2.6 ± 3.1, 1–16^a^	
Dermatological diseases	24 (2.3)	5.5, 7.1 ± 5.2, 0–16^a^		13 (54.2)	11 (45.8)		104, 152.5 ± 177.8, 1.7–756		3, 3.6 ± 3.6, 1–15^a^	
Cysts of the jaws	23 (2.2)	14, 13.5 ± 3.1, 6–18^b^		11 (47.8)	12 (52.2)		24.1, 81 ± 87.1, 8.1–234		1, 2.5 ± 7, 1–35^c^	
Amelogenesis imperfecta and odontodysplasia	22 (2.1)	13.5, 13.5 ± 3.8, 6–18^a^		12 (54.5)	10 (45.5)		24.4, 87.6 ± 116.2, 8.1–445		3, 3.9 ± 2.8, 1–12^a^	
Diseases with somatic and cognitive developmental abnormalities	20 (1.9)	7.50, 8.4 ± 4.3, 2–16^a^		10 (50)	10 (50)		22.6, 98.6 ± 157.8, 1.7–542		2, 2.1 ± 1.5, 1–7^a^	
Diseases of brain development and intellectual disability	20 (1.9)	10.5, 9.8 ± 4.3, 1–16^a^		9 (45)	11 (55)		8.1, 35.6 ± 76, 1.7–312		1, 1.7 ± 0.9, 1–4^a^	
Renal and urological diseases	20 (1.9)	8, 7.6 ± 4.5, 2–16^a^		8 (40)	12 (60)		19.3, 54.1 ± 122.2, 1.7–514		2, 2.2 ± 1.2, 1–5^a^	
Neuromuscular diseases	18 (1.7)	8.5, 8.8 ± 4.1, 1–16^a^		2 (11.1)	16 (88.9)		14.2, 65.9 ± 161.3, 1.7–699		1, 1.8 ± 1, 1–4^a^	
Neurodegenerative diseases	17 (1.6)	9, 9.6 ± 4.2, 3–16^a^		9 (54.9)	8 (47.1)		24.4, 35.3 ± 42.4, 1.7–149		1, 1.5 ± 0.8, 1–4^a^	
Heart diseases	15 (1.4)	6, 6.33 ± 4.9, 0–17^a^		32 (55.2)	26 (44.8)		30.2, 111.5 ± 157.9, 1.7–511		1, 2.2 ± 2, 1–8^a^	
Endocrine diseases	7 (0.7)	4, 5.2 ± 5.7, 1–17^a^		5 (71.4)	2 (28.6)		8.1, 96.7 ± 192.3, 1.7–526		2, 3.2 ± 3.3, 1–10^a^	
Diseases of the gastrointestinal system	4 (0.4)	8.5, 8.5 ± 5.6, 2–15^a^		1 (25)	3 (75)		30.7, 49.8 ± 63.6, 1.7–136		1, 1.2 ± 0.5, 1–2^a^	
Ophthalmological diseases	2 (0.2)	5, 5 ± 5.6, 1–9^b^		1 (50)	1 (50)		2, 2 ± 0.4, 1.7–2.3		1, 1 ± 0, 1–1^d^	

*Note:* Superscript letters indicate groupings based on Dunn's *post hoc* analysis. Services sharing the same letter do not differ significantly (*p* ≥ 0.05), while those with different letters show a statistically significant difference (*p* < 0.05).

^‡^ Median, mean ± SD, and range.

* Kruskal–Wallis test.

** Chi‐squared test.

Patients from the South/Southwest and Triângulo Mineiro/Alto Paranaíba regions initiated dental care at significantly younger ages (mean: 3.6 and 3.4, respectively). Longer average travel distances were found in Campo das Vertentes and Central Mineira (mean: 192.0 and 191.0 km, respectively) and the shortest mean distance was found in metropolitan Belo Horizonte (30.4 km). The Vale do Mucuri region registered the highest mean number of annual appointments (*n* = 5.0), followed by the South/Southwest region (*n* = 4.0). Significant regional disparities were found within the state of Minas Gerais with regard to age at first appointment (*p* < 0.001), distance travelled to specialised centres (*p* < 0.001) and mean annual number of visits (*p* = 0.002) (Table 4).

**TABLE 4:** Comparison among regions of Minas Gerais regarding age at first consultation, travel distance, and annual number of consultations (*n* = 1056*).

Region of the state of Minas Gerais	*n* (%)	Age (years) at first consultation^‡^	*p* **	Sex, *n* (%)		*p* ***	Distance to specialised centre in km^‡^ ^,^ *	*p* **	Annual visits^‡^	*p* **
				Female	Male					
Campo das Vertentes*	23 (2.2)	5, 5.6 ± 4.8, 0–16^b^	**< 0.001**	10 (43.2)	13 (56.6)	0.861	179, 192 ± 46.6, 136–269^b^	**< 0.001**	1, 2.9 ± 3,3, 1–14^a^	**0.002**
Central Mineira	30 (2.8)	9.5, 8.3 ± 4.2, 0–16^a^		13 (43.3)	17 (56.7)		179, 191 ± 36.4, 148–286^b^		1, 1.7 ± 1.2, 1–5^a^	
Jequitinhonha	25 (2.4)	9, 9 ± 5.2, 0–17^a^		8 (32)	17 (68)		543, 526.3 ± 168.1, 231–826^b^		1, 2.1 ± 1.7, 1–7^a^	
Belo Horizonte	718 (67.9)	9, 9.21 ± 4.9, 0–18^a^		326 (45.4)	392 (54.6)		20.3, 30.4 ± 37,1, 1.7–273^c^		1, 2.2 ± 2.5, 1–36^a^	
Northwest	3 (0.3)	11, 10.3 ± 2, 8–12^a^		1 (33.3)	2 (66.7)		495, 510 ± 136.6, 382–654^a^		2, 2 ± 1, 1–3^a^	
North	64 (6.1)	9, 9.3 ± 5.1, 0–18^a^		36 (56.3)	28 (43.8)		177, 252 ± 222.3, 25–793^a^		2, 2.2 ± 1.4, 1–7^a^	
West	34 (3.2)	9, 9.2 ± 4.7, 0–17^a^		15 (44.1)	19 (55.9)		148.5, 161.7 ± 50.8, 87.4–262^a^		2, 3.7 ± 4, 1–19^a^	
South/Southwest	37 (3.5)	2, 3.6 ± 4.7, 0–17^b^		16 (43.2)	21 (56.8)		335, 341.1 ± 280.8, 23–389^bc^		2, 4 ± 4.6, 1–20^a^	
Triângulo Mineiro/Alto Paranaíba	24 (2.3)	2, 3.4 ± 3.7, 0–12^b^		11 (45.8)	13 (54.2)		5.6, 59.5 ± 156.4, 5.6–612^bc^		3, 2.6 ± 1.2, 1–6^b^	
Vale do Mucuri	12 (1.1)	9.5, 9.4 ± 3.9, 3–15^a^		6 (50)	6 (50)		515, 514.9 ± 55.3, 445–603^a^		2, 5 ± 6.4, 1–20^a^	
Vale do Rio Doce	44 (4.2)	5, 5.9 ± 4.33, 0–17^b^		17 (38.6)	27 (61.4)		297, 290 ± 90.7, 55.9–625^a^		1, 2 ± 1.8, 1–10^a^	
Zona da Mata	42 (4)	6, 6.6 ± 4.6, 0–18^a^		19 (45.2)	23 (54.8)		275, 281 ± 72.3, 176–510^a^		1, 2 ± 1.7, 1–8^a^	

*Note:* Superscript letters indicate groupings based on Dunn's post hoc analysis. Services sharing the same letter do not differ significantly (*p* ≥ 0.05), whereas those with different letters show a statistically significant difference (*p* < 0.05).

^**‡**^ Median, mean ± SD, and range.

* One patient treated at Unimontes resided in a municipality in Bahia, located in northeastern Brazil.

** Kruskal–Wallis test.

*** Chi‐squared test.

## 4 Discussion

Data from this study demonstrates the structural and systemic barriers that children and adolescents living with RDs face in gaining access to specialised dental care. Despite public health policies designed to promote equity, persistent disparities remain, particularly due to the centralization of specialised centres and the limited integration of oral health in RD care pathways [13, 14, 15]. This scenario exemplifies how centralised healthcare structures restrict access for individuals residing in distant regions [21, 22] and also aligns with biopolitical frameworks, wherein healthcare infrastructure and access are unevenly distributed as mechanisms of governance and control [22]. This pattern is not unique to Brazil; it reflects broader global trends, in which specialised RD centres are predominantly concentrated in tertiary centres, creating geographic bottlenecks that disproportionately burden patients in underserved areas [2, 23].

In the present study, individuals with RDs frequently travelled long distances to access specialised treatment and often experienced delays in receiving their first dental appointment. However, geographical proximity alone did not ensure better access. In some remote regions, patients began care earlier and maintained more regular follow‐up than those residing in the capital city. This shows that distance, while relevant, is not the sole determinant of the use of specialised dental centres. Factors such as institutional organisation, referral efficiency and local centre capacity may exert a more decisive influence [11, 15]. Thus, access should be understood not merely in spatial terms, but as a systemic phenomenon shaped by the ways in which care is structured and operationalised within the broader health network [15, 24].

These disparities are further compounded by fragmented care pathways that impose substantial logistical and emotional burdens on families [5, 24, 25, 26]. Drawing on Mol's concept of the ontology of care [27], it can be argued that such fragmentation does not result from incidental gaps, but rather from systemic configurations in which access to dental care is contingent, reactive and unevenly distributed. Despite its diagnostic and functional relevance, oral health remains poorly integrated into the continuum of RD care [8, 9, 15, 28]. Many RDs involve early craniofacial signs that could facilitate a timely diagnosis and intervention, yet dentistry is rarely included in early screening protocols. This failure to incorporate oral health into routine RD assessments leads to delays in appropriate referrals, promotes reliance on reactive and often complex interventions, and further exacerbates the long‐term burden of disease [14, 15]. Marked differences in the use of specialised dental centres were also found across disease categories: individuals with syndromes affecting the craniofacial complex had the highest average number of annual dental visits (4.8 visits per year), whereas those with odontogenic tumours and neurodegenerative diseases averaged only 1.5 visits, suggesting underutilization even in groups with recognised oral and maxillofacial involvement. As noted by Okunev et al. [29], these patterns exemplify a reactive logic in which oral care is sought primarily in response to complications, rather than being embedded within a preventive framework.

As highlighted by Friedlander et al. [19], the systematic identification of oral phenotypes in RDs is essential to enhancing diagnostic accuracy and improving patient outcomes through timely referral to specialised oral healthcare centres. The need for long‐distance travel to access specialised centres raises serious concerns about the continuity of care, adherence to follow‐up, and the exacerbation of disparities in oral health outcomes, particularly among individuals living in rural or underserved regions [24, 25, 26]. Although centralised systems facilitate access to high‐complexity care, such systems paradoxically constrain early and preventive interventions, which are crucial components for mitigating the progression of disease. The present study demonstrates that most individuals with RDs experience considerable delays in gaining access to oral healthcare, often attending their first dental appointment only in pre‐adolescence, i.e., well beyond the optimal window for preventive action. Notably, patients from the Triângulo Mineiro region, who resided nearest to specialised centres (median distance: 5.6 km), reported the highest median number of annual appointments (three visits), demonstrating the importance of efficient local centre organisation. In contrast, residents of the capital city, despite the closer geographic proximity (median distance: 20.3 km), had the same low median number of annual visits (*n* = 1.2) as patients from the remote Jequitinhonha region (median distance exceeding 540 km). These findings indicate that geographic distance alone does not fully account for disparities in centre utilisation. This is particularly concerning in conditions such as Fanconi anaemia and dyskeratosis congenita, which are associated with a high risk of oral cancer and require early detection and sustained vigilance [30]. Collectively, these data underscore the urgent need for a decentralised RD care model in which specialised regional hubs serve as intermediaries between primary care and tertiary centres, thereby expanding access without compromising quality [2, 19, 31].

Interestingly, most individuals in this study resided in metropolitan Belo Horizonte (state capital), which is partially explained by the migration of families in search of specialised treatment [5, 23]. This internal patient mobility reflects the centralization of RD care, particularly at the UFMG hospital, which is a national reference centre. However, the concentration of patients in a single facility gives rise to systemic inefficiencies, such as long waiting times, diagnostic delays and substantial travel burdens [26, 32]. This study also analysed centres that, although not officially designated RD reference centres, offer specialised care through collaborations with academic institutions. The aim was to map the distribution of patients with RDs treated at these facilities and assess the capacity to provide specialised care. Such institutions fulfil a critical yet often underrecognized role in expanding access to RD centres and could serve as strategic components in a decentralised healthcare model. In this context, the low number of RD cases reported in regions such as Triângulo Mineiro/Alto Paranaíba may not reflect the true epidemiological burden, but rather result from restrictive selection criteria, underscoring the need for broader data collection strategies to capture the full extent of RD distribution.

Haematological diseases accounted for the majority of cases analysed, as these patients received dental care at the UFMG hospital during the course of their medical treatment. This predominance may be partially explained by the strong integration between haematology and hospital‐based dental centres, which facilitates systematic referrals for patients requiring specialised oral healthcare. As many haematological conditions, such as leukaemias and bone marrow disorders, require rigorous oral management to prevent complications during systemic therapy, dental assessments are often embedded within multidisciplinary hospital protocols. At the same time, this pattern reveals structural limitations within the broader healthcare network, particularly the lack of specialised training among primary care dentists, which contributes to an over‐reliance on hospital‐based specialists for managing RD‐related oral manifestations [5, 15, 17, 19]. As a result, access to oral healthcare in primary care settings is often bypassed, with individuals receiving care only when hospital‐based centres are available.

Expanding this discussion to a global context, disparities in RD care between low‐ and high‐income countries reveal the stratification of access to medical centres. A comparative policy analysis by Ng et al. [33] demonstrated that high‐income countries, such as Australia, the United Kingdom and the United States, have adopted reimbursement models, risk‐sharing agreements and public‐private partnerships to enhance access to orphan drugs. Despite these mechanisms, financial barriers persist and, in some cases, even high‐income countries depend on donation‐based funding, thereby limiting equitable access [34]. In low‐income settings (e.g., Latin America), such medications are often unavailable and, when available, remain economically prohibitive [2]. Moreover, the very classification of RD is shaped by epistemic and geopolitical contexts; conditions considered rare in affluent regions may be more common in resource‐limited settings due to genetic, environmental and infrastructural factors [2]. Such disparities go beyond logistical obstacles, reflecting entrenched ideological frameworks that influence what diseases receive research funding, pharmaceutical development and policy prioritisation [2, 33, 34]. Thus, rarity is not merely an intrinsic attribute of a disease, but a constructed category shaped by economic and political forces that determine who receives care and who remains excluded from biomedical assistance [2].

Individuals with RDs who have oral phenotypes, such as dental anomalies in ectodermal dysplasias or odontogenic cysts/tumours in individuals with syndromes, may exhibit important diagnostic characteristics [31, 35]. Integrating oral health into clinical phenotyping can enhance diagnostic accuracy, enabling earlier identification and intervention [28, 35]. Thus, oral healthcare is essential, given the functional importance of the oral cavity and the risks associated with poor oral hygiene, including the need for invasive treatments and increased financial burden. Beyond physical health, oral health profoundly affects self‐esteem and psychosocial well‐being, as aesthetic concerns and pain can impair both emotional and social functioning [10, 36]. The involvement of dentists strengthens future perspectives in RD care by contributing to the advancement of molecular diagnostics and the implementation of early, collaborative interventions with medical specialists [37]. It also supports the development of specialised care, such as orthodontic treatment, which can improve oral functioning and enhance the quality of life and self‐esteem of individuals living with RDs [10, 36, 38]. Coordinated, interdisciplinary strategies, as adopted in select European RD networks, have shown promise in reducing healthcare disparities by incorporating routine oral health assessments into standard care pathways, thereby promoting earlier interventions and better outcomes [19].

The limitations of the present study should be acknowledged. Approximately 12.8% of the initially identified cases were excluded due to missing or incomplete data, particularly regarding diagnostic confirmation or treatment history. This proportion reflects potential record‐keeping weaknesses and may introduce selection bias. Moreover, the study population was restricted to children and adolescents treated at five specialised dental centres, which may limit the generalizability of findings to broader healthcare settings, particularly primary care services or underserved regions with no referral infrastructure. These drawbacks underscore the need for improvement in documentation practices and more integrated data systems within the Brazilian universal healthcare system. Nonetheless, the strengths of this study include a diverse sample from across the state of Minas Gerais and a standardised data collection protocol, which enhance the reliability of the findings regarding access to dental care for paediatric patients with RDs in one of the largest and most populous states of Brazil. As a strategic direction, the establishment of regional specialised centres linked to national reference hospitals, which is a model that has been successfully implemented in European countries [19, 31], could promote the decentralisation of RD care in Brazil. Achieving such integration, however, requires sustained investment in professional training, as many dentists remain insufficiently prepared to manage this population [39]. Lastly, beyond logistical challenges, seeking care for RDs remains an emotionally and physically demanding process, often compounded by structural inequalities [10, 40]. Patients and caregivers routinely face long wait times, geographic displacement and administrative barriers, further intensifying their burden [40].

In summary, individuals with RDs in Brazil typically have their first dental appointment only in pre‐adolescence, have few annual follow‐up visits and must travel long distances to gain access to specialised care. The distribution of both patients and reference centres is markedly uneven, with a disproportionate concentration of centres in the state capital. These findings underscore the urgent need to reorganise the distribution of centres, promote early oral health interventions and strengthen interdisciplinary strategies that integrate oral healthcare into comprehensive RD management frameworks.

## Author Contributions

H.V.P., R.M.S.S. and G.G.F.G.F. were responsible for data collection and drafting the manuscript. F.S.O., D.R.B.M., K.R.J.C., A.V.M.V.S. and I.F.T.M. contributed to data collection and revision of the manuscript. B.R.B., H.L.B.S. and B.M.J. assisted with data curation. L.R.F.S., K.D.R. and K.L.S.M. participated in data collection and methodological supervision. C.R.M.L., B.P.J.F., D.V.T., S.M. and J.A.A.A. contributed to study design, data interpretation and drafting of the manuscript. A.C.B.‐O., H.M.‐J. and T.A.S. conceived the study, supervised the research, and contributed to the interpretation of the results. All authors provided critical feedback, helped refine the research and analysis, and contributed to the final version of the manuscript.

## Ethics Statement

Ethical approval was obtained from the Human Research Ethics Committees of the Universidade Federal de Minas Gerais (no. 63051222.0.0000.5149), Universidade Professor Edson Antônio Velano (no. 67606822.1.0000.5143) and Universidade Federal de Uberlândia (no. 36046920.0.0000.5152). The study was conducted in accordance with the Declaration of Helsinki.

## Conflicts of Interest

The authors declare no conflicts of interest.

## Supporting information


**File S1:** Definitions and coding for study variables.

**File S2:** Distribution of 244 rare diseases (*n* = 1057).

**File S3:** Distribution of rare diseases, mean annual visits, age at first consultation, sex, specialised centre, residential region of patients and mean distance travelled (*n* = 1057).

## Data Availability

Data upon which this study is based is available from the authors upon request.
