# Supplementary material for: Mapping Dental Care for Children and Adolescents With Rare Diseases: A Brazilian Multicentre Study
Source: Community Dent Oral Epidemiol. 2025 Oct 3;54(2):163–73. doi: 10.1111/cdoe.70029 (PMC13001003; doi:10.1111/cdoe.70029)
Supplement: Supplementary file 1 — File S1: Definitions and coding for study variables. [file CDOE-54-163-s003.docx]

**Supplementary File 1.** Definitions and coding for study variables

| **Category** | **Variable** | **Definition** | **Measurement/format** | **Coding/response options** | **Source** |
| --- | --- | --- | --- | --- | --- |
| **Demographic** | Date of birth | Date on which the patient was born | Date (DD/MM/YYYY) | – | Medical or dental records |
|  | Date of first dental consultation | Date when the patient first accessed dental services | Date (DD/MM/YYYY) | – | Dental records |
|  | Sex | Biological sex assigned at birth | Categorical | 1. Male  2. Female | Medical or dental records |
|  | Residential address | Registered residence of the patient | Categorical | - City of Minas Gerais  **- Regional division:**  1. *Campo das Vertentes*  2. *Central Mineira*  3. *Jequitinhonha*  4. Belo Horizonte region  5. Northwest of Minas Gerais  6. North of Minas Gerais  7. West of Minas Gerais  8. South and Southwest of Minas Gerais  9. *Triângulo Mineiro* and *Alto Paranaíba*  10. *Vale do Mucuri*  11. *Vale do Rio Doce*  12. *Zona da Mata* | - City: Medical or dental records  - Regional division: defined by the Brazilian Institute of Geography and Statistics (IBGE) (https://www.mg.gov.br/pagina/geografia) |
| **Clinical** | Rare disease diagnosis | Confirmed diagnosis of a rare disease | Categorical | - Name of rare disease - ORPHA code - **Categories:**  1. Hematological diseases 2. Genetic diseases 3. Autoimmune and autoinflammatory diseases 4. Bone diseases 5. Non-odontogenic tumors (benign and malignant) 6. Syndromes with oral and maxillofacial manifestations 7. Disease with motor/cognitive expression of the central nervous system 8. Odontogenic tumors (benign and malignant) 9. Liver diseases 10. Metabolic diseases 11. Vascular diseases 12. Dermatological diseases 13. Cysts of the jaws 14. Amelogenesis imperfecta and odontodysplasia 15. Diseases with somatic and cognitive developmental abnormalities 16. Diseases of brain development and intellectual disability 17. Renal and urological diseases 18. Neuromuscular diseases 19. Neurodegenerative diseases 20. Heart diseases 21. Endocrine diseases 22. Diseases of the gastrointestinal system 23. Ophthalmological diseases | - Name of rare disease: medical or dental records  - ORPHA code: orphanet database https://www.orpha.net  - Disease categories: based on an adapted version of Friedlander et al. (2022) which classifies conditions according to the primary physiological system involded |
|  | Age at first dental consultation | Patient’s age at first dental consultation | Continuous (years) | 0, 1, 2, ... | Dental records |
| **Healthcare access** | Number of annual dental visits | Average number of documented dental visits per year | Discrete numeric (count) | 0, 1, 2, ... | Dental records |
|  | Travel distance to center | Estimated distance from residence to specialized dental center (shortest route) | Continuous (km) | Calculated using Google Maps API | Google Maps API, based on home address <https://www.google.com.br/maps/preview> |
