# Supplementary material for: Mapping Dental Care for Children and Adolescents With Rare Diseases: A Brazilian Multicentre Study
Source: Community Dent Oral Epidemiol. 2025 Oct 3;54(2):163–73. doi: 10.1111/cdoe.70029 (PMC13001003; doi:10.1111/cdoe.70029)
Supplement: Supplementary file 2 — File S2: Distribution of 244 rare diseases (n = 1057). [file CDOE-54-163-s001.docx]

**Supplementary File** **2.** Distribution of 244 rare diseases (*n*=1,057)

| **Group studied** | ***n*** | **%** | **Rare diseases** | **Orpha Code** | ***n*** | **%** |
| --- | --- | --- | --- | --- | --- | --- |
| **Hematological diseases** | 410 | 38.9 | Idiopathic Aplastic Anemia/Bone Marrow Aplasia | 88 | 24 | 2.3 |
|  |  |  | Fanconi Anemia | 84 | 25 | 2.4 |
|  |  |  | Autoimmune Hemolytic Anemia | 98375 | 11 | 1 |
|  |  |  | Microcytic Anemia | 1047 | 2 | 0.2 |
|  |  |  | Sickle Cell Anemia | 232 | 12 | 1.1 |
|  |  |  | Factor VII Deficiency | 327 | 1 | 0.1 |
|  |  |  | Castleman Disease | 160 | 1 | 0.1 |
|  |  |  | Hereditary Spherocytosis | 822 | 9 | 0.9 |
|  |  |  | Unspecified Acute Leukemia | 98835 | 5 | 0.5 |
|  |  |  | Acute Lymphoblastic Leukemia (ALL) | 513 | 161 | 15.3 |
|  |  |  | Chronic Lymphocytic Leukemia (CLL) | 67038 | 1 | 0.1 |
|  |  |  | Acute Myeloid Leukemia (AML) | 519 | 42 | 4 |
|  |  |  | Chronic Myeloid Leukemia (CML) | 521 | 7 | 0.7 |
|  |  |  | Juvenile Myelomonocytic Leukemia | 86834 | 1 | 0.1 |
|  |  |  | Promyelocytic Leukemia (APL) | 520 | 1 | 0.1 |
|  |  |  | Burkitt Lymphoma (NHL) | 543 | 11 | 1 |
|  |  |  | Hodgkin Lymphoma | 98293 | 23 | 2.2 |
|  |  |  | Non-Hodgkin Lymphoma | 547 | 15 | 1.4 |
|  |  |  | Mastocytosis | 98292 | 1 | 0.1 |
|  |  |  | Prothrombin Gene Mutation | 325 | 1 | 0.1 |
|  |  |  | Cyclic Neutropenia | 2686 | 2 | 0.2 |
|  |  |  | Idiopathic Thrombocytopenic Purpura (ITP) | 3002 | 30 | 2.8 |
|  |  |  | Thrombotic Thrombocytopenic Purpura | 54057 | 1 | 0.1 |
|  |  |  | Diamond-Blackfan Syndrome | 124 | 5 | 0.5 |
|  |  |  | Chediak-Higashi Syndrome | 167 | 4 | 0.4 |
|  |  |  | Myelodysplastic Syndrome | 52688 | 9 | 0.9 |
|  |  |  | Wiskott-Aldrich Syndrome | 906 | 2 | 0.2 |
|  |  |  | Hereditary Thrombocytosis | 71493 | 2 | 0.2 |
|  |  |  | Thrombophilia | 217454 | 1 | 0.1 |
| **Genetic diseases** | 94 | 9 | Adrenoleukodystrophy | 43 | 1 | 0.1 |
|  |  |  | Neurofibromatosis Type I | 636 | 8 | 0.8 |
|  |  |  | Neurofibromatosis Type II | 637 | 1 | 0.1 |
|  |  |  | Deletion of the Long Arm of Chromosome 6 | 262047 | 2 | 0.2 |
|  |  |  | Arthrogryposis-Renal-Cholestasis Syndrome | 109007 | 1 | 0.1 |
|  |  |  | Beckwith-Wiedemann Syndrome | 116 | 2 | 0.2 |
|  |  |  | CHARGE Syndrome | 138 | 3 | 0.3 |
|  |  |  | Goldenhar Syndrome | 141132 | 8 | 0.8 |
|  |  |  | Oculo-Auriculo-Vertebral Dysplasia | 828 | 1 | 0.1 |
|  |  |  | Cornelia de Lange Syndrome | 199 | 6 | 0.6 |
|  |  |  | Edwards Syndrome | 3380 | 1 | 0.1 |
|  |  |  | Ellis-van Creveld Syndrome | 289 | 1 | 0.1 |
|  |  |  | Escobar Syndrome | 2990 | 1 | 0.1 |
|  |  |  | Evans Syndrome | 1959 | 2 | 0.2 |
|  |  |  | Phosphoinositide 3-Kinase Delta Syndrome | 397596 | 1 | 0.1 |
|  |  |  | Gardner Syndrome | 79665 | 1 | 0.1 |
|  |  |  | Glass Syndrome (SATB2 gene) | 576278 | 1 | 0.1 |
|  |  |  | Griscelli Syndrome | 381 | 1 | 0.1 |
|  |  |  | Hoyeraal-Hreidarsson Syndrome | 3322 | 1 | 0.1 |
|  |  |  | Jacobsen Syndrome | 2308 | 1 | 0.1 |
|  |  |  | Jarcho-Levin Syndrome | 2311 | 1 | 0.1 |
|  |  |  | Kabuki Syndrome | 2322 | 1 | 0.1 |
|  |  |  | Primary Ciliary Dyskinesia (Kartagener Syndrome) | 244 | 1 | 0.1 |
|  |  |  | Klippel-Trenaunay Syndrome | 90308 | 1 | 0.1 |
|  |  |  | Kostmann Syndrome | 99749 | 2 | 0.2 |
|  |  |  | Laron Syndrome | 633 | 2 | 0.2 |
|  |  |  | Leigh Syndrome | 506 | 1 | 0.1 |
|  |  |  | Lowe Syndrome | 534 | 2 | 0.2 |
|  |  |  | Marfan Syndrome | 558 | 2 | 0.2 |
|  |  |  | Pallister-Killian Syndrome | 884 | 1 | 0.1 |
|  |  |  | Patau Syndrome | 3378 | 1 | 0.1 |
|  |  |  | Pearson Syndrome | 699 | 1 | 0.1 |
|  |  |  | Pelizaeus-Merzbacher Syndrome | 702 | 2 | 0.2 |
|  |  |  | Phelan-McDermid Syndrome | 48652 | 1 | 0.1 |
|  |  |  | Proteus Syndrome | 744 | 1 | 0.1 |
|  |  |  | Prune Belly Syndrome | 2970 | 3 | 0.3 |
|  |  |  | Rubinstein-Taybi Syndrome | 783 | 3 | 0.3 |
|  |  |  | Sotos Syndrome | 821 | 1 | 0.1 |
|  |  |  | Stevens-Johnson Syndrome | 36426 | 1 | 0.1 |
|  |  |  | TAR Syndrome (Thrombocytopenia-Absent Radius) | 3320 | 3 | 0.3 |
|  |  |  | VATER or VACTERL Syndrome | 887 | 1 | 0.1 |
|  |  |  | Waardenburg Syndrome | 3440 | 2 | 0.2 |
|  |  |  | Williams Syndrome | 904 | 5 | 0.5 |
|  |  |  | Wolf-Hirschhorn Syndrome | 280 | 2 | 0.2 |
|  |  |  | Ring Chromosome 10 Syndrome | 1438 | 1 | 0.1 |
|  |  |  | Ring Chromosome 18 Syndrome | 1442 | 1 | 0.1 |
|  |  |  | KBG Syndrome | 2332 | 1 | 0.1 |
|  |  |  | L1CAM Syndrome | 275543 | 1 | 0.1 |
|  |  |  | Orofacial Digital Syndrome | 140997 | 2 | 0.2 |
|  |  |  | PFAPA Syndrome (Periodic Fever, Aphthous Stomatitis, Pharyngitis, and Adenitis) | 42642 | 1 | 0.1 |
|  |  |  | PHACE Syndrome (Posterior Fossa anomalies, Hemangioma, Arterial anomalies, Cardiac defects, Eye abnormalities) | 42775 | 1 | 0.1 |
|  |  |  | Popliteal Pterygium Syndrome | 1300 | 1 | 0.1 |
| **Autoimmnue and autoinflammatory diseases** | 64 | 6.1 | Hereditary Angioedema | 91378 | 1 | 0.1 |
|  |  |  | Takayasu Arteritis | 3287 | 1 | 0.1 |
|  |  |  | Juvenile Idiopathic Arthritis | 92 | 1 | 0.1 |
|  |  |  | Oligoarticular Juvenile Idiopathic Arthritis | 85410 | 1 | 0,1 |
|  |  |  | Deficiency of the Interleukin-1 Receptor Antagonist (DIRA) | 210115 | 1 | 0.1 |
|  |  |  | Juvenile Dermatomyositis | 93672 | 1 | 0.1 |
|  |  |  | Behçet's Disease | 117 | 2 | 0.2 |
|  |  |  | Graft-versus-Host Disease | 39812 | 2 | 0.2 |
|  |  |  | Chronic Granulomatous Disease | 379 | 1 | 0.1 |
|  |  |  | Angioneurotic Edema | 658 | 1 | 0.1 |
|  |  |  | Rheumatic Fever | 3099 | 1 | 0.1 |
|  |  |  | Langerhans Cell Histiocytosis | 389 | 14 | 1.3 |
|  |  |  | Severe Combined Immunodeficiency (SCID) | 183660 | 4 | 0.4 |
|  |  |  | Primary Immunodeficiency | 101997 | 5 | 0.5 |
|  |  |  | Lichen Planus | 254367 | 5 | 0.5 |
|  |  |  | Systemic Lupus Erythematosus | 536 | 10 | 0.9 |
|  |  |  | Mucosal Pemphigus Vulgaris | 704 | 1 | 0.1 |
|  |  |  | Blau Syndrome | 90340 | 1 | 0.1 |
|  |  |  | Cogan Syndrome | 1467 | 1 | 0.1 |
|  |  |  | Antiphospholipid Antibody Syndrome | 80 | 1 | 0.1 |
|  |  |  | Hemophagocytic Syndrome | 158032 | 7 | 0.7 |
|  |  |  | Autoimmune Lymphoproliferative Syndrome (ALPS) | 3261 | 2 | 0.2 |
| **Bone diseases** | 60 | 5.7 | Metaphyseal Chondrodysplasia | 175 | 1 | 0.1 |
|  |  |  | Cleidocranial Dysplasia | 1452 | 2 | 0.2 |
|  |  |  | Blount Disease | 2768 | 1 | 0.1 |
|  |  |  | Progressive Ossifying Myositis | 337 | 1 | 0.1 |
|  |  |  | Microcephalic Osteodysplastic Primordial Dwarfism Type 2 | 2637 | 1 | 0.1 |
|  |  |  | Osteogenesis Imperfecta | 666 | 29 | 2.7 |
|  |  |  | Osteosarcoma | 668 | 18 | 1.7 |
|  |  |  | Rickets Type I | 289157 | 1 | 0.1 |
|  |  |  | Apert Syndrome | 87 | 4 | 0.4 |
|  |  |  | Tibial Aplasia-Ectrodactyly Syndrome | 3329 | 1 | 0.1 |
|  |  |  | Ehlers-Danlos Syndrome | 98249 | 1 | 0.1 |
| **Non-odontogenic tumors (benign and malignant)** | 58 | 5.5 | Pilocytic Astrocytoma | 251612 | 5 | 0.5 |
|  |  |  | Craniopharyngioma | 54595 | 1 | 0.1 |
|  |  |  | Ganglioneuroma | 251992 | 2 | 0.2 |
|  |  |  | Meningioma | 2495 | 1 | 0.1 |
|  |  |  | Myofibromatosis | 2591 | 1 | 0.1 |
|  |  |  | Schwannoma/Fibroblastoma | 252164 | 5 | 0.5 |
|  |  |  | Pleomorphic Adenoma | 454821 | 3 | 0.3 |
|  |  |  | Xanthoastrocytoma | 251607 | 1 | 0.1 |
|  |  |  | Neurofibroma | 252183 | 4 | 0.4 |
|  |  |  | Anaplastic Ependymoma | 251646 | 2 | 0.2 |
|  |  |  | Glioma | 182067 | 2 | 0.2 |
|  |  |  | Medulloblastoma | 616 | 8 | 0.8 |
|  |  |  | Neuroblastoma | 635 | 5 | 0.5 |
|  |  |  | Rhabdomyosarcoma | 780 | 2 | 0.2 |
|  |  |  | Retinoblastoma | 790 | 3 | 0.3 |
|  |  |  | Ewing Sarcoma | 319 | 3 | 0.3 |
|  |  |  | Histiocytic Sarcoma | 86896 | 1 | 0.1 |
|  |  |  | Clear Cell Renal Sarcoma | 457246 | 1 | 0.1 |
|  |  |  | Synovial Sarcoma | 3273 | 1 | 0.1 |
|  |  |  | Nasopharyngeal Squamous Cell Carcinoma | 494547 | 2 | 0.2 |
|  |  |  | Choroid Plexus Carcinoma | 251899 | 1 | 0.1 |
|  |  |  | Adrenal Gland Carcinoma | 1501 | 2 | 0.2 |
|  |  |  | Neurofibrosarcoma | 3148 | 1 | 0.1 |
|  |  |  | Ovarian Teratoma | 398987 | 1 | 0.1 |
| **Syndromes with oral and maxillofacial manifestations** | 51 | 4.7 | Cherubism | 184 | 1 | 0.1 |
|  |  |  | Weyers Acrofacial Dysostosis | 952 | 1 | 0.1 |
|  |  |  | Crouzon Syndrome | 207 | 1 | 0.1 |
|  |  |  | Moebius Syndrome | 570 | 6 | 0.6 |
|  |  |  | Pierre Robin Sequence | 718 | 33 | 3.1 |
|  |  |  | Treacher Collins Syndrome | 861 | 3 | 0.3 |
|  |  |  | Van der Woude Syndrome | 888 | 5 | 0.5 |
|  |  |  | Solitary Median Maxillary Central Incisor Syndrome | 280200 | 1 | 0.1 |
| **Disease with motor/cognitive expression of the central nervous system** | 30 | 2.7 | Rasmussen Encephalitis | 1929 | 1 | 0.1 |
|  |  |  | Hypoxic-Ischemic Encephalopathy | 137577 | 1 | 0.1 |
|  |  |  | Schizencephaly | 799 | 2 | 0.2 |
|  |  |  | Hydrocephalus | 2185 | 10 | 0.9 |
|  |  |  | Microcephaly | 2512 | 1 | 0.1 |
|  |  |  | Microcephaly Associated with Zika Virus | 448237 | 2 | 0.2 |
|  |  |  | Myelomeningocele | 93969 | 6 | 0.6 |
|  |  |  | Dandy-Walker Syndrome | 217 | 4 | 0.4 |
|  |  |  | Coffin-Lowry Syndrome | 192 | 1 | 0.1 |
|  |  |  | Fetal alcohol syndrome | 1915 | 2 | 0.2 |
| **Odontogenic tumors (benign and malignant)** | 26 | 2.5 | Ameloblastoma | 314419 | 8 | 0.8 |
|  |  |  | Congenital Epulis | 157826 | 1 | 0.1 |
|  |  |  | Ossifying Fibroma / Central Ossifying Fibroma / Peripheral Ossifying Fibroma | 435329 | 16 | 1.5 |
|  |  |  | Peripheral Cemento-Ossifying Fibroma | 314425 | 1 | 0.1 |
| **Liver diseases** | 24 | 2.3 | Biliary Atresia | 30391 | 1 | 0.1 |
|  |  |  | Primary Sclerosing Cholangitis | 171 | 6 | 0.6 |
|  |  |  | Alpha-1 Antitrypsin Deficiency | 60 | 3 | 0.3 |
|  |  |  | Caroli Disease | 53035 | 2 | 0.2 |
|  |  |  | Wilson Disease | 905 | 1 | 0.1 |
|  |  |  | Isolated Congenital Hepatic Fibrosis | 485426 | 4 | 0.4 |
|  |  |  | Autoimmune Hepatitis | 2137 | 5 | 0.5 |
|  |  |  | Budd-Chiari Syndrome | 131 | 2 | 0.2 |
| **Metabolic diseases** | 24 | 2.3 | Methylmalonic Acidemia | 293355 | 1 | 0.1 |
|  |  |  | Propionic Acidemia | 35 | 1 | 0.1 |
|  |  |  | Glutaric Aciduria | 25 | 2 | 0.2 |
|  |  |  | Gaucher Disease | 355 | 6 | 0.6 |
|  |  |  | Cystic Fibrosis | 586 | 3 | 0.3 |
|  |  |  | Glycogen Storage Disease Type I (Von Gierke Disease) | 364 | 2 | 0.2 |
|  |  |  | Infantile Systemic Hyalinosis | 2176 | 1 | 0.1 |
|  |  |  | Lipoid Proteinosis | 530 | 1 | 0.1 |
|  |  |  | Mucopolysaccharidoses | 79213 | 7 | 0.7 |
| **Vascular diseases** | 24 | 2.3 | Congenital Kaposiform Hemangioendothelioma | 2122 | 1 | 0.1 |
|  |  |  | Congenital Hemangioma | 458775 | 13 | 1.2 |
|  |  |  | Lymphangioma | 2415 | 6 | 0.6 |
|  |  |  | Pulmonary Lymphangiomatosis | 2414 | 1 | 0.1 |
|  |  |  | Portal Vein Thrombosis | 854 | 1 | 0.1 |
|  |  |  | Cerebral Venous Thrombosis | 329217 | 1 | 0.1 |
|  |  |  | Facial Arteriovenous Malformation | 156230 | 1 | 0.1 |
| **Dermatological Diseases** | 24 | 2.3 | Ectodermal Dysplasia | 79373 | 11 | 1 |
|  |  |  | Hypohidrotic Ectodermal Dysplasia | 189 | 2 | 0.2 |
|  |  |  | Epidermolysis Bullosa | 303 | 8 | 0.8 |
|  |  |  | Bloch-Sulzberger Syndrome (also known as Incontinentia Pigmenti) | 464 | 1 | 0.1 |
|  |  |  | Cowden Syndrome | 201 | 1 | 0.1 |
|  |  |  | Xeroderma Pigmentosum | 910 | 1 | 0.1 |
| **Cysts of the jaws** | 23 | 2.2 | Odontogenic Keratocyst | 447777 | 12 | 1.1 |
|  |  |  | Aneurysmal Bone Cyst/Idiopathic Bone Cavity/Simple Bone Cyst/Traumatic Cyst | 480553 | 11 | 1 |
| **Amelogenesis imperfecta and odontodysplasia** | 22 | 2.1 | Amelogenesis Imperfecta | 88661 | 20 | 1.9 |
|  |  |  | Odontodysplasia | 83450 | 2 | 0.2 |
| **Diseases with somatic and cognitive developmental abnormalities** | 20 | 1.9 | Neuropsychomotor Developmental Delay | 404448 | 2 | 0.2 |
|  |  |  | Cri-du-Chat Syndrome | 281 | 1 | 0.1 |
|  |  |  | Prader-Willi Syndrome | 739 | 4 | 0.4 |
|  |  |  | DiGeorge Syndrome | 567 | 4 | 0.4 |
|  |  |  | Duplication Syndrome 15q11q13 | 238446 | 1 | 0.1 |
|  |  |  | Noonan Syndrome | 648 | 8 | 0.8 |
| **Diseases of brain development and intellectual disability** | 20 | 1.9 | Rett Syndrome | 778 | 3 | 0.3 |
|  |  |  | West Syndrome | 3451 | 15 | 1.4 |
|  |  |  | Fragile X Syndrome | 908 | 2 | 0.2 |
| **Renal and urological diseases** | 20 | 1.9 | Cystinosis | 213 | 1 | 0.1 |
|  |  |  | Bilateral Renal Dysplasia | 93173 | 2 | 0.2 |
|  |  |  | Autosomal Recessive Polycystic Kidney Disease | 731 | 1 | 0.1 |
|  |  |  | Renal Hypoplasia | 93101 | 1 | 0.1 |
|  |  |  | IgA Nephropathy | 34145 | 1 | 0.1 |
|  |  |  | Nephrotic Syndrome/Focal Segmental Glomerulosclerosis | 357502 | 9 | 0.9 |
|  |  |  | Wilms Tumor | 654 | 3 | 0.3 |
|  |  |  | Posterior Urethral Valve (PUV) | 93110 | 1 | 0.1 |
|  |  |  | Fanconi Syndrome | 3337 | 1 | 0.1 |
| **Neuromuscular diseases** | 18 | 1.7 | Congenital Multiple Arthrogryposis | 1037 | 1 | 0.1 |
|  |  |  | Congenital Muscular Dystrophy | 97242 | 1 | 0.1 |
|  |  |  | Duchenne Muscular Dystrophy | 98896 | 7 | 0.7 |
|  |  |  | Systemic Sclerosis | 90291 | 1 | 0.1 |
|  |  |  | Tuberous Sclerosis | 805 | 4 | 0.4 |
|  |  |  | Congenital Myasthenia | 590 | 3 | 0.3 |
|  |  |  | Congenital Myopathy | 97245 | 1 | 0.1 |
| **Neurodegenerative diseases** | 17 | 1.6 | Spinal Muscular Atrophy | 70 | 6 | 0.6 |
|  |  |  | Steinert's Myotonic Dystrophy | 273 | 3 | 0.3 |
|  |  |  | Mitochondrial Encephalopathy | 550 | 1 | 0.1 |
|  |  |  | Ceroid Lipofuscinosis | 216 | 1 | 0.1 |
|  |  |  | Andermann Syndrome | 1496 | 1 | 0.1 |
|  |  |  | Angelman Syndrome | 72 | 3 | 0.3 |
|  |  |  | Cockayne Syndrome | 191 | 1 | 0.1 |
|  |  |  | Tay-Sachs Syndrome | 845 | 1 | 0.1 |
| **Heart diseases** | 15 | 1.4 | Congenital Atrioventricular Block | 60041 | 2 | 0.2 |
|  |  |  | Dilated Cardiomyopathy | 217604 | 1 | 0.1 |
|  |  |  | Double Outlet Right Ventricle | 3426 | 1 | 0.1 |
|  |  |  | Atrioventricular Septal Defect | 1329 | 1 | 0.1 |
|  |  |  | Endomyocardial Fibrosis (Davies Disease) | 75565 | 1 | 0.1 |
|  |  |  | Pulmonary and Supra-aortic Stenosis | 3192 | 1 | 0.1 |
|  |  |  | Pulmonary Valve Stenosis | 3189 | 1 | 0.1 |
|  |  |  | Congenital Mitral Insufficiency | 95465 | 1 | 0.1 |
|  |  |  | Tetralogy of Fallot | 3303 | 6 | 0.6 |
| **Endocrine diseases** | 7 | 0.7 | Congenital Hyperinsulinism | 657 | 1 | 0.1 |
|  |  |  | Berardinelli-Seip Syndrome | 528 | 1 | 0.1 |
|  |  |  | Turner Syndrome | 881 | 5 | 0.5 |
| **Diseases of the gastrointestinal system** | 4 | 0.4 | Eosinophilic Gastroenteropathy | 2070 | 2 | 0.2 |
|  |  |  | Tuberculosis of the Intestine, Peritoneum, and Mesenteric Lymph Nodes (Gastric Tuberculosis) | 645859 | 1 | 0.1 |
|  |  |  | Omphalocele | 660 | 1 | 0.1 |
| **Ophthalmological diseases** | 2 | 0.2 | Genetic Pediatric Glaucoma | 359 | 1 | 0.1 |
|  |  |  | Blepharophimosis Syndrome | 126 | 1 | 0.1 |
| **Total** | 1,057 | 100 |  |  | 1,057 | 100 |
