# Supplementary material for: Mapping Dental Care for Children and Adolescents With Rare Diseases: A Brazilian Multicentre Study
Source: Community Dent Oral Epidemiol. 2025 Oct 3;54(2):163–73. doi: 10.1111/cdoe.70029 (PMC13001003; doi:10.1111/cdoe.70029)
Supplement: Supplementary file 3 — File S3: Distribution of rare diseases, mean annual visits, age at first consultation, sex, specialised centre, residential region of patients and mean distance travelled (n = 1057). [file CDOE-54-163-s002.docx]

**Supplementary File 3.** Distribution of rare diseases, mean annual visits, age at first consultation, sex, specialized center, residential region of patients, and mean distance traveled (*n*=1,057)

| **Rare diseases** | **Individuals *n* (%)** | **Mean annual visits (median, mean ± SD, and range)** | **Age (years) at first consultation (median, mean ± SD, and range)** | **Distance to specialized**  **center in km (median, mean ± SD, and range)** | **Sex, *n* (%)** | **Specialized center** | ***n* (%)** | **Residential region** | ***n* (%)** | **Distance to specialized center in km (mean ± SD)** |
| --- | --- | --- | --- | --- | --- | --- | --- | --- | --- | --- |
| **Hematological diseases** | 410 (38.9) | 1, 2.4 ± 3.3, 1–36 | 9, 8.7 ± 4.6, 0–18 | 40.9, 111.6 ± 149.4, 1.7–826 | Male: 241 (58.8) | HC-UFMG^a^ | 409 (99.8) | Campo das Vertentes | 10 (2.4) | 181.3 ± 31 |
|  |  |  |  |  |  |  |  | Central Mineira | 19 (4.6) | 193.7 ± 36 |
|  |  |  |  |  |  |  |  | Jequitinhonha | 9 (2.2) | 530.6 ± 189 |
|  |  |  |  |  |  | SD-UFMG^b^ | 1 (0.2) | Belo Horizonte region | 291 (71) | 35.3 ± 40.3 |
|  |  |  |  |  |  |  |  | Northwest of Minas Gerais | 1 (0.2) | 382 |
|  |  |  |  |  |  | SD-UFU^c^ | 0 | North of Minas Gerais | 13 (3.2) | 496 ± 146.5 |
|  |  |  |  |  |  |  |  | West of Minas Gerais | 17 (4.1) | 154.9 ± 41.7 |
|  |  |  |  |  | Female: 169 (41.2) | Unimontes^d^ | 0 | South and Southwest of Minas Gerais | 1 (0.2) | 297 |
|  |  |  |  |  |  |  |  | Triângulo Mineiro and Alto Paranaíba | 1 (0.2) | 406 |
|  |  |  |  |  |  | Pro-sorriso^e^ | 0 | Vale do Mucuri | 7 (1.7) | 511.4 ± 59 |
|  |  |  |  |  |  |  |  | Vale do Rio Doce | 23 (5.6) | 282 ± 76.6 |
|  |  |  |  |  |  |  |  | Zona da Mata | 18 (4.4) | 276.9 ± 35.6 |
| **Genetic diseases** | 94 (9) | 2, 2 ± 1.6, 1–9 | 6, 6.78 ± 5.1, 0–17 | 24.4, 104.2 ± 152.9, 1.7–607 | Male: 44 (46.8) | HC-UFMG^a^ | 45 (47.4) | Campo das Vertentes | 1 (1.1) | 244 |
|  |  |  |  |  |  |  |  | Central Mineira | 3 (3.2) | 185.3 ± 22.1 |
|  |  |  |  |  |  |  |  | Jequitinhonha | 4 (4.2) | 486.5 ± 172.7 |
|  |  |  |  |  |  | SD-UFMG^b^ | 29 (31.6) | Belo Horizonte region | 54 (57.9) | 24 ± 30.4 |
|  |  |  |  |  |  |  |  | Northwest of Minas Gerais | 0 | – |
|  |  |  |  |  |  | SD-UFU^c^ | 8 (8.4) | North of Minas Gerais | 0 | – |
|  |  |  |  |  |  |  |  | West of Minas Gerais | 1 (1.1) | 135 |
|  |  |  |  |  | Female: 50 (53.2) | Unimontes^d^ | 1 (1.1) | South and Southwest of Minas Gerais | 9 (9.5) | 95.5 ± 71.4 |
|  |  |  |  |  |  |  |  | Triângulo Mineiro and Alto Paranaíba | 8 (8.4) | 5.6 ± 0 |
|  |  |  |  |  |  | Pro-sorriso^e^ | 11 (11.6) | Vale do Mucuri | 1 (1.1) | 603 |
|  |  |  |  |  |  |  |  | Vale do Rio Doce | 6 (6.3) | 333.1 ± 29.5 |
|  |  |  |  |  |  |  |  | Zona da Mata | 7 (7.4) | 312.8 ± 121.7 |
| **Autoimmune and autoinflammatory diseases** | 64 (6.1) | 1, 2.3 ± 4.2, 1–33 | 9, 8.8 ± 5.8, 0–17 | 36.8, 99.1 ± 149.4, 1.7–670 | Male: 33 (51.6) | HC-UFMG^a^ | 53 (82.8) | Campo das Vertentes | 1 (1.6) | 246 |
|  |  |  |  |  |  |  |  | Central Mineira | 3 (4.7) | 171 ± 6.9 |
|  |  |  |  |  |  |  |  | Jequitinhonha | 1 (1.6) | 670 |
|  |  |  |  |  |  | SD-UFMG^b^ | 8 (12.5) | Belo Horizonte region | 47 (73.4) | 32.4 ± 36.1 |
|  |  |  |  |  |  |  |  | Northwest of Minas Gerais | 0 | – |
|  |  |  |  |  | Female: 31 (48.4) | SD-UFU^c^ | 0 | North of Minas Gerais | 5 (7.8) | 275.1 ± 294 |
|  |  |  |  |  |  |  |  | West of Minas Gerais | 0 | – |
|  |  |  |  |  |  |  |  | South and Southwest of Minas Gerais | 1 (1.6) | 356 |
|  |  |  |  |  |  | Unimontes^d^ | 3 (4.7) | Triângulo Mineiro and Alto Paranaíba | 0 | – |
|  |  |  |  |  |  |  |  | Vale do Mucuri | 0 | – |
|  |  |  |  |  |  | Pro-sorriso^e^ | 0 | Vale do Rio Doce | 4 (6.3) | 274.5 ± 74.5 |
|  |  |  |  |  |  |  |  | Zona da Mata | 2 (3.1) | 281 ± 48 |
| **Bone diseases** | 60 (5.7) | 2, 3.3 ± 4.4, 1–25 | 9, 9.3 ± 5.3, 0–18 | 27.3, 78.9 ± 119.9, 1.7–496 | Male: 34 (56.7) | HC-UFMG^a^ | 31 (51.7) | Campo das Vertentes | 0 | – |
|  |  |  |  |  |  |  |  | Central Mineira | 2 (3.3) | 184.5 ± 31.8 |
|  |  |  |  |  |  |  |  | Jequitinhonha | 0 | – |
|  |  |  |  |  |  | SD-UFMG^b^ | 20 (33.3) | Belo Horizonte region | 40 (66.7) | 23.6 ± 27.7 |
|  |  |  |  |  |  |  |  | Northwest of Minas Gerais | 0 | – |
|  |  |  |  |  |  | SD-UFU^c^ | 4 (6.7) | North of Minas Gerais | 7 (11.7) | 242.4 ± 194 |
|  |  |  |  |  | Female: 26 (43.3) |  |  | West of Minas Gerais | 3 (5) | 148.4 ± 61.3 |
|  |  |  |  |  |  |  |  | South and Southwest of Minas Gerais | 2 (3.3) | 225.6 ± 224 |
|  |  |  |  |  |  | Unimontes^d^ | 4 (6.7) | Triângulo Mineiro and Alto Paranaíba | 4 (6.7) | 41.5 ± 55.16 |
|  |  |  |  |  |  |  |  | Vale do Mucuri | 0 | – |
|  |  |  |  |  |  | Pro-sorriso^e^ | 1 (1.7) | Vale do Rio Doce | 1 (1.7) | 358 |
|  |  |  |  |  |  |  |  | Zona da Mata | 1 (1.7) | 306 |
| **Non-odontogenic tumors (benign and malignant)** | 58 (5.5) | 1, 1.9 ± 2, 1–14 | 7, 8.1 ± 4.6, 0–18 | 24.1, 91.6 ± 158.6, 1.7–826 | Male: 26 (44.8) | HC-UFMG^a^ | 44 (75.9) | Campo das Vertentes | 2 (3.4) | 209 ± 52.3 |
|  |  |  |  |  |  |  |  | Central Mineira | 0 | – |
|  |  |  |  |  |  |  |  | Jequitinhonha | 3 (5.2) | 560.6 ± 264 |
|  |  |  |  |  |  | SD-UFMG^b^ | 12 (20.7) | Belo Horizonte region | 42 (72.4) | 24.4 ± 28.8 |
|  |  |  |  |  |  |  |  | Northwest of Minas Gerais | 0 | – |
|  |  |  |  |  |  | SD-UFU^c^ | 0 | North of Minas Gerais | 3 (5.2) | 242.8 ± 307.9 |
|  |  |  |  |  | Female: 32 (55.2) |  |  | West of Minas Gerais | 5 (8.6) | 121.7 ± 40.4 |
|  |  |  |  |  |  |  |  | South and Southwest of Minas Gerais | 0 | – |
|  |  |  |  |  |  | Unimontes^d^ | 2 (3.4) | Triângulo Mineiro and Alto Paranaíba | 0 | – |
|  |  |  |  |  |  |  |  | Vale do Mucuri | 0 | – |
|  |  |  |  |  |  | Pro-sorriso^e^ | 0 | Vale do Rio Doce | 0 | – |
|  |  |  |  |  |  |  |  | Zona da Mata | 3 (5.2) | 284 ± 19.2 |
| **Syndromes with oral and maxillofacial manifestations** | 51 (4.7) | 3, 4.8 ± 5.2, 1–26 | 1, 3.56 ± 4.8, 0–16 | 58.7, 117.7 ± 144.2, 1.7–654 | Male: 29 (56) | HC-UFMG^a^ | 1 (2) | Campo das Vertentes | 6 (12) | 203.3 ± 69.4 |
|  |  |  |  |  |  |  |  | Central Mineira | 1 (2) | 286 |
|  |  |  |  |  |  |  |  | Jequitinhonha | 0 | – |
|  |  |  |  |  |  | SD-UFMG^b^ | 19 (38) | Belo Horizonte region | 19 (37) | 23.1 ± 26.1 |
|  |  |  |  |  |  |  |  | Northwest of Minas Gerais | 1 (2) | 654 |
|  |  |  |  |  |  | SD-UFU^c^ | 2 (4) | North of Minas Gerais | 2 (4) | 78.2 ± 37.9 |
|  |  |  |  |  | Female: 21 (44) |  |  | West of Minas Gerais | 3 (6) | 230 ± 32.9 |
|  |  |  |  |  |  |  |  | South and Southwest of Minas Gerais | 14 (27) | 88.5 ± 60.3 |
|  |  |  |  |  |  | Unimontes^d^ | 0 | Triângulo Mineiro and Alto Paranaíba | 2 (4) | 5.6 ± 0 |
|  |  |  |  |  |  |  |  | Vale do Mucuri | 0 | – |
|  |  |  |  |  |  | Pro-sorriso^e^ | 27 (54) | Vale do Rio Doce | 2 (4) | 419 ± 291.3 |
|  |  |  |  |  |  |  |  | Zona da Mata | 1 (2) | 377 |
| **Disease with motor/cognitive expression of the central nervous system** | 30 (2.7) | 1, 1.8 ± 1.49, 1–7 | 6, 6.75 ± 3.8, 1–13 | 22, 103.1 ± 193, 1.7–645 | Male: 16 (57.1) | HC-UFMG^a^ | 23 (75) | Campo das Vertentes | 0 | – |
|  |  |  |  |  |  |  |  | Central Mineira | 0 | – |
|  |  |  |  |  |  |  |  | Jequitinhonha | 2 (7.1) | 694 ± 57.9 |
|  |  |  |  |  |  | SD-UFMG^b^ | 6 (21.4) | Belo Horizonte region | 25 (82.1) | 18.6 ± 15.6 |
|  |  |  |  |  |  |  |  | Northwest of Minas Gerais | 0 | – |
|  |  |  |  |  |  | SD-UFU^c^ | 0 | North of Minas Gerais | 1 (3.6) | 563 |
|  |  |  |  |  | Female: 14 (42.9) |  |  | West of Minas Gerais | 0 | – |
|  |  |  |  |  |  |  |  | South and Southwest of Minas Gerais | 0 | – |
|  |  |  |  |  |  | Unimontes^d^ | 0 | Triângulo Mineiro and Alto Paranaíba | 0 | – |
|  |  |  |  |  |  |  |  | Vale do Mucuri | 0 | – |
|  |  |  |  |  |  | Pro-sorriso^e^ | 1 (3.6) | Vale do Rio Doce | 1 (3.6) | 343 |
|  |  |  |  |  |  |  |  | Zona da Mata | 1 (3.6) | 473 |
| **Odontogenic tumors (benign and malignant)** | 26 (2.5) | 1, 1.5 ± 1.4, 1–6 | 14.5, 14.4 ± 3.4, 2–18 | 20.3, 61.8 ± 69.7, 2.5–218 | Male: 16 (61.5) | HC-UFMG^a^ | 1 (3.8) | Campo das Vertentes | 1 (3.8) | 161 |
|  |  |  |  |  |  |  |  | Central Mineira | 1 (3.8) | 148 |
|  |  |  |  |  |  |  |  | Jequitinhonha | 0 | – |
|  |  |  |  |  |  | SD-UFMG^b^ | 17 (65.4) | Belo Horizonte region | 17 (65.4) | 35.4 ± 41.5 |
|  |  |  |  |  |  |  |  | Northwest of Minas Gerais | 0 | - |
|  |  |  |  |  |  | SD-UFU^c^ | 0 | North of Minas Gerais | 7 (26.9) | 99.6 ± 95.5 |
|  |  |  |  |  | Female: 10 (38.5) |  |  | West of Minas Gerais | 0 | – |
|  |  |  |  |  |  |  |  | South and Southwest of Minas Gerais | 0 | – |
|  |  |  |  |  |  | Unimontes^d^ | 8 (30.8) | Triângulo Mineiro and Alto Paranaíba | 0 | – |
|  |  |  |  |  |  |  |  | Vale do Mucuri | 0 | - |
|  |  |  |  |  |  | Pro-sorriso^e^ | 0 | Vale do Rio Doce | 0 | – |
|  |  |  |  |  |  |  |  | Zona da Mata | 0 | – |
| **Liver diseases** | 24 (2.3) | 2, 2.5 ± 2.1, 1–8 | 10.5, 9.7 ± 5.2, 0–18 | 36.8, 134.5 ± 190.9, 1.7–645 | Male: 11 (45.8) | HC-UFMG^a^ | 0 | Campo das Vertentes | 0 | – |
|  |  |  |  |  |  |  |  | Central Mineira | 0 | – |
|  |  |  |  |  |  |  |  | Jequitinhonha | 2 (8.3) | 594 ± 72.1 |
|  |  |  |  |  |  | SD-UFMG^b^ | 24 (100) | Belo Horizonte region | 17 (70.8) | 31.12 ± 40.1 |
|  |  |  |  |  |  |  |  | Northwest of Minas Gerais | 0 | – |
|  |  |  |  |  |  | SD-UFU^c^ | 0 | North of Minas Gerais | 0 | – |
|  |  |  |  |  |  |  |  | West of Minas Gerais | 1 (4.2) | 203 |
|  |  |  |  |  | Female: 13 (54.2) |  |  | South and Southwest of Minas Gerais | 0 | – |
|  |  |  |  |  |  | Unimontes^d^ | 0 | Triângulo Mineiro and Alto Paranaíba | 0 | – |
|  |  |  |  |  |  |  |  | Vale do Mucuri | 1 (4.2) | 514 |
|  |  |  |  |  |  | Pro-sorriso^e^ | 0 | Vale do Rio Doce | 0 | – |
|  |  |  |  |  |  |  |  | Zona da Mata | 3 (12.5) | 265 ± 43.2 |
| **Metabolic diseases** | 24 (2.3) | 2, 2 ± 1.6, 1–8 | 8.5, 9 ± 5.13, 0–18 | 20.9, 48.9 ± 62.5, 1.7–248 | Male: 16 (66.7) | HC-UFMG^a^ | 11 (45.8) | Campo das Vertentes | 0 | – |
|  |  |  |  |  |  |  |  | Central Mineira | 0 | – |
|  |  |  |  |  |  |  |  | Jequitinhonha | 0 | – |
|  |  |  |  |  |  | SD-UFMG^b^ | 11 (45.8) | Belo Horizonte region | 21 (87.5) | 42.3 ± 48.7 |
|  |  |  |  |  |  |  |  | Northwest of Minas Gerais | 0 | – |
|  |  |  |  |  |  | SD-UFU^c^ | 1 (4.2) | North of Minas Gerais | 0 | – |
|  |  |  |  |  |  |  |  | West of Minas Gerais | 0 | – |
|  |  |  |  |  | Female: 8 (33.3) |  |  | South and Southwest of Minas Gerais | 1 (4.2) | 31 |
|  |  |  |  |  |  | Unimontes^d^ | 0 | Triângulo Mineiro and Alto Paranaíba | 1 (4.2) | 5.6 |
|  |  |  |  |  |  |  |  | Vale do Mucuri | 0 | – |
|  |  |  |  |  |  | Pro-sorriso^e^ | 1 (4.2) | Vale do Rio Doce | 1 (4.2) | 248 |
|  |  |  |  |  |  |  |  | Zona da Mata | 0 | – |
| **Vascular diseases** | 24 (2.3) | 2, 2.6 ± 3.1, 1–16 | 8.5, 8.4 ± 4.3, 1–16 | 5.3, 87.6 ± 162.7, 1.7–612 | Male: 10 (41.7) | HC-UFMG^a^ | 10 (41.7) | Campo das Vertentes | 0 | – |
|  |  |  |  |  |  |  |  | Central Mineira | 0 | – |
|  |  |  |  |  |  |  |  | Jequitinhonha | 0 | – |
|  |  |  |  |  |  | SD-UFMG^b^ | 1 (4.2) | Belo Horizonte region | 9 (37.5) | 27.2 ± 34.4 |
|  |  |  |  |  |  |  |  | Northwest of Minas Gerais | 1 (4.2) | 495 |
|  |  |  |  |  |  | SD-UFU^c^ | 0 | North of Minas Gerais | 13 (54.2) | 57.7 ± 97.5 |
|  |  |  |  |  |  |  |  | West of Minas Gerais | 0 | – |
|  |  |  |  |  | Female: 14 (58.3) |  |  | South and Southwest of Minas Gerais | 0 | – |
|  |  |  |  |  |  | Unimontes^d^ | 13 (54.2) | Triângulo Mineiro and Alto Paranaíba | 1 (4.2) | 612 |
|  |  |  |  |  |  |  |  | Vale do Mucuri | 0 | – |
|  |  |  |  |  |  | Pro-sorriso^e^ | 0 | Vale do Rio Doce | 0 | – |
|  |  |  |  |  |  |  |  | Zona da Mata | 0 | – |
| **Dermatological diseases** | 24 (2.3) | 3, 3.6 ± 3.6, 1–15 | 5.5, 7.1 ± 5.2, 0–16 | 104, 152.5 ± 177.8, 1.7–756 | Male: 11 (45.8) | HC-UFMG^a^ | 7 (29.2) | Campo das Vertentes | 0 | – |
|  |  |  |  |  |  |  |  | Central Mineira | 0 | – |
|  |  |  |  |  |  |  |  | Jequitinhonha | 1 (4.2) | 231 |
|  |  |  |  |  |  | SD-UFMG^b^ | 2 (8.3) | Belo Horizonte region | 6 (25) | 48.5 ± 57.6 |
|  |  |  |  |  |  |  |  | Northwest of Minas Gerais | 0 | – |
|  |  |  |  |  |  | SD-UFU^c^ | 1 (4.2) | North of Minas Gerais | 9 (37.5) | 125.4 ± 124.9 |
|  |  |  |  |  |  |  |  | West of Minas Gerais | 1 (4.2) | 182 |
|  |  |  |  |  | Female: 13 (54.2) |  |  | South and Southwest of Minas Gerais | 5 (20.8) | 213.2 ± 160.7 |
|  |  |  |  |  |  | Unimontes^d^ | 11 (45.8) | Triângulo Mineiro and Alto Paranaíba | 1 (4.2) | 5.6 |
|  |  |  |  |  |  |  |  | Vale do Mucuri | 0 | – |
|  |  |  |  |  |  | Pro-sorriso^e^ | 3 (12.5) | Vale do Rio Doce | 0 | – |
|  |  |  |  |  |  |  |  | Zona da Mata | 0 | – |
|  |  |  |  |  |  |  |  | Bahia^*^ | 1 (4.2) | 756 |
| **Cysts of the jaws** | 23 (2.2) | 1, 2.5 ± 7, 1–35 | 14, 13.5 ± 3.1, 6–18 | 24.1, 81 ± 87.1, 8.1–234 | Male: 12 (52.2) | HC-UFMG^a^ | 1 (4.3) | Campo das Vertentes | 0 | – |
|  |  |  |  |  |  |  |  | Central Mineira | 1 (4.3) | 190 |
|  |  |  |  |  |  |  |  | Jequitinhonha | 0 | – |
|  |  |  |  |  |  | SD-UFMG^b^ | 19 (82.6) | Belo Horizonte region | 15 (65.2) | 25.2 ± 40.6 |
|  |  |  |  |  |  |  |  | Northwest of Minas Gerais | 0 | – |
|  |  |  |  |  |  | SD-UFU^c^ | 0 | North of Minas Gerais | 3 (13) | 169.3 ± 17.6 |
|  |  |  |  |  |  |  |  | West of Minas Gerais | 2 (8.7) | 170 ± 70.7 |
|  |  |  |  |  | Female: 11 (47.8) |  |  | South and Southwest of Minas Gerais | 0 | – |
|  |  |  |  |  |  | Unimontes^d^ | 3 (13) | Triângulo Mineiro and Alto Paranaíba | 0 | – |
|  |  |  |  |  |  |  |  | Vale do Mucuri | 0 | – |
|  |  |  |  |  |  | Pro-sorriso^e^ | 0 | Vale do Rio Doce | 1 (4.3) | 213 |
|  |  |  |  |  |  |  |  | Zona da Mata | 1 (4.3) | 234 |
| **Amelogenesis imperfecta and odontodysplasia** | 22 (2.1) | 3, 3.9 ± 2.8, 1–12 | 13.5, 13.5 ± 3.8, 6–18 | 24.4, 87.6 ± 116.2, 8.1–445 | Male: 10 (45.5) | HC-UFMG^a^ | 0 | Campo das Vertentes | 0 | – |
|  |  |  |  |  |  |  |  | Central Mineira | 0 | – |
|  |  |  |  |  |  |  |  | Jequitinhonha | 0 | – |
|  |  |  |  |  |  | SD-UFMG^b^ | 22 (100) | Belo Horizonte region | 17 (77.3) | 34.9 ± 35.8 |
|  |  |  |  |  |  |  |  | Northwest of Minas Gerais | 0 | – |
|  |  |  |  |  |  | SD-UFU^c^ | 0 | North of Minas Gerais | 0 | – |
|  |  |  |  |  |  |  |  | West of Minas Gerais | 0 | – |
|  |  |  |  |  | Female: 12 (54.5) |  |  | South and Southwest of Minas Gerais | 0 | – |
|  |  |  |  |  |  | Unimontes^d^ | 0 | Triângulo Mineiro and Alto Paranaíba | 0 | – |
|  |  |  |  |  |  |  |  | Vale do Mucuri | 1 (4.5) | 445 |
|  |  |  |  |  |  | Pro-sorriso^e^ | 0 | Vale do Rio Doce | 0 | – |
|  |  |  |  |  |  |  |  | Zona da Mata | 4 (18.2) | 222.2 ± 72.8 |
| **Diseases with somatic and cognitive developmental abnormalities** | 20 (1.9) | 2, 2.1 ± 1.5, 1–7 | 7.50, 8.4 ± 4.3, 2–16 | 22.6, 98.6 ± 157.8, 1.7–542 | Male: 10 (50) | HC-UFMG^a^ | 14 (70) | Campo das Vertentes | 0 | – |
|  |  |  |  |  |  |  |  | Central Mineira | 0 | – |
|  |  |  |  |  |  |  |  | Jequitinhonha | 2 (10) | 471.5 ± 99.7 |
|  |  |  |  |  |  | SD-UFMG^b^ | 5 (25) | Belo Horizonte region | 14 (70) | 26.3 ± 35.3 |
|  |  |  |  |  |  |  |  | Northwest of Minas Gerais | 0 | – |
|  |  |  |  |  |  | SD-UFU^c^ | 1 (5) | North of Minas Gerais | 0 | – |
|  |  |  |  |  |  |  |  | West of Minas Gerais | 0 | – |
|  |  |  |  |  | Female: 10 (50) |  |  | South and Southwest of Minas Gerais | 1 (5) | 350 |
|  |  |  |  |  |  | Unimontes^d^ | 0 | Triângulo Mineiro and Alto Paranaíba | 1 (5) | 5.6 |
|  |  |  |  |  |  |  |  | Vale do Mucuri | 0 | – |
|  |  |  |  |  |  | Pro-sorriso^e^ | 0 | Vale do Rio Doce | 2 (10) | 152.4 ± 136.5 |
|  |  |  |  |  |  |  |  | Zona da Mata | 0 | – |
| **Diseases of brain development and intellectual disability** | 20 (1.9) | 1, 1.7 ± 0.9, 1–4 | 10.5, 9.8 ± 4.3, 1–16 | 8.1, 35.6 ± 76, 1.7–312 | Male: 11 (55) | HC-UFMG^a^ | 1 (20) | Campo das Vertentes | 0 | – |
|  |  |  |  |  |  |  |  | Central Mineira | 0 | – |
|  |  |  |  |  |  |  |  | Jequitinhonha | 0 | – |
|  |  |  |  |  |  | SD-UFMG^b^ | 16 (80) | Belo Horizonte region | 18 (90) | 12.2 ± 12.6 |
|  |  |  |  |  |  |  |  | Northwest of Minas | 0 | – |
|  |  |  |  |  |  | SD-UFU^c^ | 0 | North of Minas Gerais | 0 | – |
|  |  |  |  |  |  |  |  | West of Minas Gerais | 0 | – |
|  |  |  |  |  | Female: 9 (45) |  |  | South and Southwest of Minas Gerais | 0 | – |
|  |  |  |  |  |  | Unimontes^d^ | 0 | Triângulo Mineiro and Alto Paranaíba | 0 | – |
|  |  |  |  |  |  |  |  | Vale do Mucuri | 0 | – |
|  |  |  |  |  |  | Pro-sorriso^e^ | 0 | Vale do Rio Doce | 1 (5) | 312 |
|  |  |  |  |  |  |  |  | Zona da Mata | 1 (5) | 180 |
| **Renal and urological diseases** | 20 (1.9) | 2, 2.2 ± 1.2, 1–5 | 8, 7.6 ± 4.5, 2–16 | 19.3, 54.1 ± 122.2, 1.7–514 | Male: 12 (60) | HC-UFMG^a^ | 19 (95) | Campo das Vertentes | 0 | – |
|  |  |  |  |  |  |  |  | Central Mineira | 0 | – |
|  |  |  |  |  |  |  |  | Jequitinhonha | 1 (5) | 514 |
|  |  |  |  |  |  | SD-UFMG^b^ | 0 | Belo Horizonte region | 17 (85) | 17.7 ± 16.7 |
|  |  |  |  |  |  |  |  | Northwest of Minas Gerais | 0 | – |
|  |  |  |  |  |  | SD-UFU^c^ | 1 (5) | North of Minas Gerais | 0 | – |
|  |  |  |  |  |  |  |  | West of Minas Gerais | 1 (5) | 262 |
|  |  |  |  |  | Female: 8 (40) |  |  | South and Southwest of Minas Gerais | 0 | – |
|  |  |  |  |  |  | Unimontes^d^ | 0 | Triângulo Mineiro and Alto Paranaíba | 1 (5) | 5.6 |
|  |  |  |  |  |  |  |  | Vale do Mucuri | 0 | – |
|  |  |  |  |  |  | Pro-sorriso^e^ | 0 | Vale do Rio Doce | 0 | – |
|  |  |  |  |  |  |  |  | Zona da Mata | 0 | – |
| **Neuromuscular diseases** | 18 (1.7) | 1, 1.8 ± 1, 1–4 | 8.5, 8.8 ± 4.1, 1–16 | 14.2, 65.9 ± 161.3, 1.7–699 | Male: 16 (88.9) | HC-UFMG^a^ | 7 (38.9) | Campo das Vertentes | 0 | – |
|  |  |  |  |  |  |  |  | Central Mineira | 0 | – |
|  |  |  |  |  |  |  |  | Jequitinhonha | 0 | – |
|  |  |  |  |  |  | SD-UFMG^b^ | 10 (55.6) | Belo Horizonte region | 16 (88.9) | 30.1 ± 34.4 |
|  |  |  |  |  |  |  |  | Northwest of Minas Gerais | 0 | – |
|  |  |  |  |  |  | SD-UFU^c^ | 1 (5.6) | North of Minas Gerais | 1 (5.6) | 699 |
|  |  |  |  |  |  |  |  | West of Minas Gerais | 0 | – |
|  |  |  |  |  | Female: 2 (11.1) |  |  | South and Southwest of Minas Gerais | 0 | – |
|  |  |  |  |  |  | Unimontes^d^ | 0 | Triângulo Mineiro and Alto Paranaíba | 1(5.6) | 5.6 |
|  |  |  |  |  |  |  |  | Vale do Mucuri | 0 | – |
|  |  |  |  |  |  | Pro-sorriso^e^ | 0 | Vale do Rio Doce | 0 | – |
|  |  |  |  |  |  |  |  | Zona da Mata | 0 | – |
| **Neurodegenerative diseases** | 17 (1.6) | 1, 1.5 ± 0.8, 1–4 | 9, 9.6 ± 4.2, 3–16 | 24.4, 35.3 ± 42.4, 1.7–149 | Male: 8 (47.1) | HC-UFMG^a^ | 5 (29.4) | Campo das Vertentes | 0 | – |
|  |  |  |  |  |  |  |  | Central Mineira | 0 | – |
|  |  |  |  |  |  |  |  | Jequitinhonha | 0 | – |
|  |  |  |  |  |  | SD-UFMG^b^ | 10 (58.8) | Belo Horizonte region | 15 (88.2) | 23.8 ± 27.4 |
|  |  |  |  |  |  |  |  | Northwest of Minas Gerais | 0 | – |
|  |  |  |  |  |  | SD-UFU^c^ | 1 (5.9) | North of Minas Gerais | 0 | – |
|  |  |  |  |  | Female: 9 (52.9) |  |  | West of Minas Gerais | 0 | – |
|  |  |  |  |  |  |  |  | South and Southwest of Minas Gerais | 1 (5.9) | 93.9 |
|  |  |  |  |  |  | Unimontes^d^ | 0 | Triângulo Mineiro and Alto Paranaíba | 1 (5.9) | 149 |
|  |  |  |  |  |  |  |  | Vale do Mucuri | 0 | – |
|  |  |  |  |  |  | Pro-sorriso^e^ | 1 (5.9) | Vale do Rio Doce | 0 | – |
|  |  |  |  |  |  |  |  | Zona da Mata | 0 | – |
| **Heart diseases** | 15 (1.4) | 1, 2.2 ± 2, 1–8 | 6, 6.33 ± 4.9, 0–17 | 30.2, 111.5 ± 157.9, 1.7–511 | Male: 6 (40) | HC-UFMG^a^ | 13 (86.7) | Campo das Vertentes | 1 (6.7) | 179 |
|  |  |  |  |  |  |  |  | Central Mineira | 0 | – |
|  |  |  |  |  |  |  |  | Jequitinhonha | 0 | – |
|  |  |  |  |  |  | SD-UFMG^b^ | 2 (13.3) | Belo Horizonte region | 11 (73.3) | 37.5 ± 79.3 |
|  |  |  |  |  |  |  |  | Northwest of Minas Gerais | 0 | – |
|  |  |  |  |  |  | SD-UFU^c^ | 0 | North of Minas Gerais | 0 | – |
|  |  |  |  |  |  |  |  | West of Minas Gerais | 0 | – |
|  |  |  |  |  | Female: 9 (60) |  |  | South and Southwest of Minas Gerais | 0 | – |
|  |  |  |  |  |  | Unimontes^d^ | 0 | Triângulo Mineiro and Alto Paranaíba | 0 | – |
|  |  |  |  |  |  |  |  | Vale do Mucuri | 1 (6.7) | 511 |
|  |  |  |  |  |  | Pro-sorriso^e^ | 0 | Vale do Rio Doce | 2 (13.3) | 286.5 ± 17.6 |
|  |  |  |  |  |  |  |  | Zona da Mata | 0 | – |
| **Endocrine diseases** | 7 (0.7) | 2, 3.2 ± 3.3, 1–10 | 4, 5.2 ± 5.7, 1–17 | 8.1, 96.7 ± 192.3, 1.7–526 | Male: 5 (71.4) | HC-UFMG^a^ | 3 (42.9) | Campo das Vertentes | 0 | – |
|  |  |  |  |  |  |  |  | Central Mineira | 0 | – |
|  |  |  |  |  |  |  |  | Jequitinhonha | 0 | – |
|  |  |  |  |  |  | SD-UFMG^b^ | 1 (14.3) | Belo Horizonte region | 3 (42.9) | 36.3 ± 54.5 |
|  |  |  |  |  |  |  |  | Northwest of Minas Gerais | 0 | – |
|  |  |  |  |  |  | SD-UFU^c^ | 2 (28.6) | North of Minas Gerais | 0 | – |
|  |  |  |  |  | Female: 5 (71.4) |  |  | West of Minas Gerais | 0 | – |
|  |  |  |  |  |  |  |  | South and Southwest of Minas Gerais | 1 (14.3) | 31 |
|  |  |  |  |  |  | Unimontes^d^ | 0 | Triângulo Mineiro and Alto Paranaíba | 2 (28.6) | 5.6 ± 0 |
|  |  |  |  |  |  |  |  | Vale do Mucuri | 1 (14.3) | 526 |
|  |  |  |  |  |  | Pro-sorriso^e^ | 1 (14.3) | Vale do Rio Doce | 0 | – |
|  |  |  |  |  |  |  |  | Zona da Mata | 0 | – |
| **Diseases of the gastrointestinal system** | 4 (0.4) | 1, 1.2 ± 0.5, 1–2 | 8.5, 8.5 ± 5.6, 2–15 | 30.7, 49.8 ± 63.6, 1.7–136 | Male: 3 (75) | HC-UFMG^a^ | 4 (100) | Campo das Vertentes | 1 (25) | 136 |
|  |  |  |  |  |  |  |  | Central Mineira | 0 | – |
|  |  |  |  |  |  |  |  | Jequitinhonha | 0 | – |
|  |  |  |  |  |  | SD-UFMG^b^ | 0 | Belo Horizonte region | 3 (75) | 21 ± 33.5 |
|  |  |  |  |  |  |  |  | Northwest of Minas Gerais | 0 | – |
|  |  |  |  |  |  | SD-UFU^c^ | 0 | North of Minas Gerais | 0 | – |
|  |  |  |  |  |  |  |  | West of Minas Gerais | 0 | – |
|  |  |  |  |  | Female: 1 (25) |  |  | South and Southwest of Minas Gerais | 0 | – |
|  |  |  |  |  |  | Unimontes^d^ | 0 | Triângulo Mineiro and Alto Paranaíba | 0 | – |
|  |  |  |  |  |  |  |  | Vale do Mucuri | 0 | – |
|  |  |  |  |  |  | Pro-sorriso^e^ | 0 | Vale do Rio Doce | 0 | – |
|  |  |  |  |  |  |  |  | Zona da Mata | 0 | – |
| **Ophthalmological diseases** | 2 (0.2) | 1, 1 ± 0, 1–1 | 5, 5 ± 5.6, 1–9 | 2, 2 ± 0.4, 1.7–2.3 | Male: 1 (50) | HC-UFMG^a^ | 1 (50) | Campo das Vertentes | 0 | – |
|  |  |  |  |  |  |  |  | Central Mineira | 0 | – |
|  |  |  |  |  |  |  |  | Jequitinhonha | 0 | – |
|  |  |  |  |  |  | SD-UFMG^b^ | 0 | Belo Horizonte region | 1 (50) | 1.7 |
|  |  |  |  |  |  |  |  | Northwest of Minas Gerais | 0 | – |
|  |  |  |  |  |  | SD-UFU^c^ | 0 | North of Minas Gerais | 0 | – |
|  |  |  |  |  |  |  |  | West of Minas Gerais | 0 | – |
|  |  |  |  |  | Female: 1 (50) |  |  | South and Southwest of Minas Gerais | 1 (50) | 2.3 |
|  |  |  |  |  |  | Unimontes^d^ | 0 | Triângulo Mineiro and Alto Paranaíba | 0 | – |
|  |  |  |  |  |  |  |  | Vale do Mucuri | 0 | – |
|  |  |  |  |  |  | Pro-sorriso^e^ | 1 (50) | Vale do Rio Doce | 0 | – |
|  |  |  |  |  |  |  |  | Zona da Mata | 0 | – |

**Note:** SD, standard deviation.

^a^Special Dental Diagnosis and Treatment Service, Hospital das Clínicas, Universidade Federal de Minas Gerais (a hospital affiliated with a public university); ^b^School of Dentistry, Universidade Federal de Minas Gerais (includes the clinic of oral medicine and pathology, the clinic for children and adolescents with disabilities, the clinic for individuals with cleft lip and palate, and the clinic for individuals with odontogenesis defects; public university); ^c^School of Dentistry, Universidade Federal de Uberlândia (public university); ^d^Oral Pathology and Oral Medicine Service, School of Dentistry, Universidade Estadual de Montes Claros (public university); and ^e^Craniofacial Anomalies Rehabilitation Center, Pro-Sorriso Center, Universidade Professor Edson Antônio Velano (a center accredited by the public health system).

^*^Bahia, northeastern region of Brazil.
